# Supplementary material for: Evaluation of a rapid lateral flow assay for the detection of taeniosis and cysticercosis at district hospital level in Tanzania: A prospective multicentre diagnostic accuracy study
Source: PLoS Negl Trop Dis. 2025 Mar 28;19(3):e0012310. doi: 10.1371/journal.pntd.0012310 (PMC11977959; doi:10.1371/journal.pntd.0012310)
Supplement: S1 Appendix — Table 1A Recruitment of cohort 1 participants from the outpatient department and mental health clinic. Table 1B. Symptoms of participants in cohort 1 during recruitment. The results are from screening questionnaires administered by local nurses. S1 Table 1C. Age and gender of patients who were selected for sampling and complete cases for the evaluation of taeniosis. S1 Table 1D. Age and gender of patients who were selected for sampling and complete cases for the evaluation of cysticercosis. (PDF) [file pntd.0012310.s001.docx]

Supporting information 1

Table A. Recruitment of cohort 1 participants from the outpatient department and mental health clinic.

|  | Recruitment location | | | Total |
| --- | --- | --- | --- | --- |
| Reason for recruitment* | Mental health clinic | Outpatient department | Missing information |  |
| Headache only | 9 | 114 | 2 | 125 |
| Epilepsy only | 152 | 13 | 4 | 169 |
| Headache and epilepsy | 261 | 43 | 3 | 307 |
| Total | 422 | 170 | 9 | 601 |

* according to screening questionnaires administered by local nurses during the recruitment of participants

Table B. Symptoms of participants in cohort 1 during recruitment. The results are from screening questionnaires administered by local nurses.

|  | Mental health clinic | Outpatient department | Missing information | Total |
| --- | --- | --- | --- | --- |
| Number of participants | 422 | 170 | 9 | 601 |
| Headache screening questionnaire |  |  |  |  |
| Are you currently suffering from severe headaches? | 129 | 153 | 4 | 286 |
| Are the current headaches keeping you from performing your work or usual activities? | 58 | 152 | 3 | 213 |
| Are your headaches increasing progressively since they have started? | 95 | 148 | 3 | 246 |
| Do you normally suffer from headaches? | 169 | 136 | 4 | 309 |
| Are your current headaches different from your usual headaches? | 82 | 140 | 3 | 225 |
| Epilepsy screening questionnaire |  |  |  |  |
| Have you ever been told that you had an episode of losing consciousness during which your arms and legs shake or stretch out? | 282 | 34 | 6 | 322 |
| During attacks of unconsciousness have you ever bitten your tongue or lost control of your bladder or bowels? | 207 | 24 | 4 | 235 |
| Have you ever had uncontrollable attacks of shaking or trembling in one arm or one leg or in the face without losing consciousness? | 194 | 33 | 5 | 232 |
| Have you ever been told that you have or had epilepsy or epileptic fits? | 287 | 30 | 5 | 322 |

Table C. Age and gender of patients who were selected for sampling and complete cases for the evaluation of taeniosis.

| **Cohort** | **Selected for sampling** | **Complete case for taeniosis evaluation** | **Number of patients** | **Mean age** | **Median age** | **IQR age** | **Minimum age** | **Maximum age** | **Number of female patients** | **Number of male patients** |
| --- | --- | --- | --- | --- | --- | --- | --- | --- | --- | --- |
| 1 | No | No | 462 | 35.0 | 32 | 22 | 10 | 82 | 247 | 215 |
| 1 | Yes | No | 19 | 37.2 | 35 | 25.5 | 20 | 60 | 10 | 9 |
| 1 | Yes | Yes | 120 | 41.0 | 38 | 25.2 | 13 | 80 | 56 | 64 |
| 2 | No | No | 1497 | 37.4 | 33 | 27 | 10 | 100 | 1044 | 453 |
| 2 | Yes | No | 50 | 39.6 | 38 | 31.5 | 10 | 80 | 27 | 23 |
| 2 | Yes | Yes | 114 | 39.7 | 39.5 | 28.8 | 12 | 79 | 75 | 39 |
| 3 | No | No | 594 | 42.8 | 40 | 32.8 | 10 | 88 | 400 | 194 |
| 3 | Yes | No | 15 | 43.5 | 38 | 26 | 17 | 81 | 11 | 4 |
| 3 | Yes | Yes | 53 | 43.9 | 40 | 29 | 14 | 85 | 34 | 19 |

Table D. Age and gender of patients who were selected for sampling and complete cases for the evaluation of cysticercosis.

| **Cohort** | **Selected for sampling** | **Complete case for taeniosis evaluation** | **Number of patients** | **Mean age** | **Median age** | **IQR age** | **Minimum age** | **Maximum age** | **Number of female patients** | **Number of male patients** |
| --- | --- | --- | --- | --- | --- | --- | --- | --- | --- | --- |
| 1 | No | No | 462 | 35.0 | 32 | 22 | 10 | 82 | 247 | 215 |
| 1 | Yes | No | 13 | 36.5 | 33 | 26 | 20 | 60 | 7 | 6 |
| 1 | Yes | Yes | 126 | 40.9 | 38.5 | 24.8 | 13 | 80 | 59 | 67 |
| 2 | No | No | 1497 | 37.4 | 33 | 27 | 10 | 100 | 1044 | 453 |
| 2 | Yes | No | 42 | 39.5 | 38 | 31.5 | 10 | 80 | 22 | 20 |
| 2 | Yes | Yes | 122 | 39.7 | 38.5 | 28.8 | 10 | 79 | 80 | 42 |
| 3 | No | No | 594 | 42.8 | 40 | 32.8 | 10 | 88 | 400 | 194 |
| 3 | Yes | No | 13 | 41.3 | 38 | 24 | 17 | 74 | 10 | 3 |
| 3 | Yes | Yes | 55 | 44.4 | 40 | 29 | 14 | 85 | 35 | 20 |
