# Supplementary material for: Evaluation of a rapid lateral flow assay for the detection of taeniosis and cysticercosis at district hospital level in Tanzania: A prospective multicentre diagnostic accuracy study
Source: PLoS Negl Trop Dis. 2025 Mar 28;19(3):e0012310. doi: 10.1371/journal.pntd.0012310 (PMC11977959; doi:10.1371/journal.pntd.0012310)
Supplement: S3 Appendix — (PDF) [file pntd.0012310.s003.pdf]

# Supporting information 2 – output of Bayesian analyses

# Evaluation of a rapid lateral flow assay for the detection of taeniosis and cysticercosis at district hospital level in Tanzania: a prospective multicentre diagnostic accuracy study

*1. Taeniosis ...... 2*
**1.1. Round 1 (original priors) ...... 2**
1.1.1. Including rES33-EITB ...... 2
1.1.2. Model 1 ...... 3
1.1.2.1. Model 2 ...... 4
1.1.2.1. Model 2B ...... 5
1.1.2.1. Model 3 ...... 6
1.1.3. Without rES33-EITB ...... 6
1.1.3.1. Model 1 ...... 7
1.1.4. Model 2 ...... 8
**1.2. Round 2 (new priors) ...... 9**
1.2.1. Priors - rationale ...... 9
1.2.2. Model output - including rES33-EITB ...... 10
1.2.3. Model output - without rES33-EITB ...... 11
**1.3. Directed acyclic graph taeniosis ...... 12**
*2. Cysticercosis ...... 12*
**2.1. Round 1 (original priors) ...... 13**
2.1.1. Model 1 ...... 14
2.1.2. Model 2 ...... 14
2.1.3. Model 3 ...... 15
**2.2. Round 2 (new priors) ...... 15**
2.2.1. Priors - rationale ...... 15
2.2.2. Model output ...... 16
**2.3. Directed acyclic graph cysticercosis ...... 17**
*3. References ...... 18*

# 1. Taeniosis

Initially the same priors were used as for the study at community level [1]. The priors and output of these models are described in section 1.1. However, since diagnostic accuracy measures differ according to the target population, and the data didn't support the priors in certain models as demonstrated by high Bayesian P values, new priors were defined, which are described in section 1.2. The models marked with an asterisk (*) have been selected as the definitive models for the manuscript.

## 1.1. Round 1 (original priors)

### 1.1.1. Including rES33-EITB

Priors (taken from [1]):

| Model | Test | LL | UL |
|---|---|---|---|
| **Model 1** | Prevalence | 0.001 | 0.05 |
| | All-sensitivity | 0.000 | 1.00 |
| | All-specificity | 0.500 | 1.00 |
| **Model 2** | Prevalence | 0.001 | 0.05 |
| | mPCR-sensitivity | 0.000 | 1.00 |
| | mPCR-specificity | 0.700 | 0.99 |
| | Other-sensitivity | 0.000 | 1.00 |
| | Other-specificity | 0.500 | 1.00 |
| **Model 2B** | Prevalence | 0.001 | 0.05 |
| | mPCR-sensitivity | 0.700 | 0.99 |
| | mPCR-specificity | 0.900 | 0.99 |
| | Other-sensitivity | 0.000 | 1.00 |
| | Other-specificity | 0.500 | 1.00 |
| **Model 3** | Prevalence | 0.001 | 0.05 |
| | mPCR-sensitivity | 0.700 | 0.99 |
| | mPCR-specificity | 0.900 | 0.99 |
| | rES33-sensitivity | 0.900 | 0.99 |
| | rES33-specificity | 0.500 | 1.00 |
| | Other-sensitivity | 0.000 | 1.00 |
| | Other-specificity | 0.500 | 1.00 |

Overview of the different models:

| Model | Variable | Cohort 1 | Cohort 2 | Cohort 3 | All |
|---|---|---|---|---|---|
| Model1 | Bayesian P | 0.675 | 0.699 | 0.443 | 0.771 |
| Model1 | Deviance | 56.9 | 53.0 | 34.8 | 75.9 |
| Model2 | Bayesian P | 0.657 | 0.597 | 0.429 | 0.729 |
| Model2 | Deviance | 55.4 | 48.857 | 33.2 | 73.4 |
| Model2B | Bayesian P | 0.657 | 0.590 | 0.425 | 0.719 |
| Model2B | Deviance | 55.6 | 48.8 | 33.3 | 73.2 |
| Model3 | Bayesian P | 0.771 | 0.638 | 0.448 | 0.871 |
| Model3 | Deviance | 56.4 | 46.9 | 31.5 | 75.8 |

### 1.1.2. Model 1

| Test | Measure | Cohort 1 | Cohort 2 | Cohort 3 | All |
|---|---|---|---|---|---|
| TS POC T | Sensitivity | 46.4 [2.4 - 96.6] | 40.3 [1 - 96] | 39.3 [1.2 - 95.7] | 41.2 [1.3 - 96] |
| TS POC T | Specificity | 98.5 [97 - 99.6] | 99.4 [98.8 - 99.8] | 99.4 [98.5 - 99.9] | 99.3 [98.8 - 99.7] |
| TS POC T | PPV | 22.7 [0.3 - 74] | 13.8 [0.1 - 66.6] | 22.8 [0.3 - 80.9] | 11.8 [0.1 - 53.6] |
| TS POC T | NPV | 99.3 [96.9 - 100] | 99.7 [97.9 - 100] | 99.5 [97.2 - 100] | 99.8 [98.5 - 100] |
| TS POC CC | Sensitivity | 51.5 [8.5 - 92.4] | 42.5 [3.8 - 90.1] | 44.9 [5.5 - 90.5] | 46.5 [5.3 - 91.5] |
| TS POC CC | Specificity | 81.7 [74.4 - 87.5] | 97.3 [96.1 - 98.4] | 96.6 [94.3 - 98.4] | 94.4 [93 - 95.6] |
| TS POC CC | PPV | 2.8 [0.1 - 10] | 4.5 [0.1 - 18.5] | 7.1 [0.1 - 31.4] | 2.2 [0 - 8.8] |
| TS POC CC | NPV | 99.3 [96.8 - 100] | 99.7 [98.1 - 100] | 99.6 [97.7 - 100] | 99.8 [98.6 - 100] |
| rES33-EITB | Sensitivity | 49.7 [14.5 - 85.4] | 48.4 [11.4 - 86.2] | 46.5 [11.2 - 84.8] | 47.3 [11.7 - 85.1] |
| rES33-EITB | sp | 93.1 [85.8 - 96.9] | 94.7 [89.2 - 98.2] | 96.9 [90 - 99.6] | 96.3 [93.2 - 98.2] |
| rES33-EITB | PPV | 7.6 [0.2 - 28.5] | 3.7 [0 - 20.7] | 11.2 [0.2 - 52] | 4.2 [0.1 - 21.6] |
| rES33-EITB | NPV | 99.4 [97.7 - 100] | 99.8 [98.5 - 100] | 99.6 [98 - 100] | 99.8 [99 - 100] |
| copro-mPCR | Sensitivity | 46 [17.7 - 77.2] | 46.3 [15.4 - 78.9] | 46.7 [16.4 - 78.7] | 45.4 [15.3 - 78.1] |
| copro-mPCR | Specificity | 95.8 [88.6 - 99] | 96.7 [92.3 - 99.2] | 96.4 [89.5 - 99.4] | 98.1 [95.7 - 99.5] |
| copro-mPCR | PPV | 12.3 [0.4 - 45.2] | 5.9 [0.1 - 33.3] | 9.5 [0.2 - 44.2] | 8 [0.1 - 39.9] |
| copro-mPCR | NPV | 99.4 [97.6 - 100] | 99.8 [98.6 - 100] | 99.6 [98 - 100] | 99.8 [98.9 - 100] |
| copro-Ag | Sensitivity | 49.1 [24.6 - 73.8] | 47.5 [21.1 - 74.4] | 48.4 [23 - 74.3] | 48.3 [22.1 - 74.7] |
| copro-Ag | Specificity | 94.3 [87.2 - 98] | 93.5 [88 - 97.3] | 93.1 [84.6 - 98] | 95.4 [92 - 97.9] |
| copro-Ag | PPV | 9.4 [0.3 - 33.6] | 3 [0 - 16.6] | 5 [0.1 - 23.9] | 3.6 [0.1 - 17.7] |
| copro-Ag | NPV | 99.4 [97.8 - 100] | 99.8 [98.6 - 100] | 99.6 [98.1 - 100] | 99.8 [99 - 100] |
| Prevalence | | 1.1 [0 - 3.7] | 0.4 [0 - 2.3] | 0.6 [0 - 3] | 0.3 [0 - 1.7] |

### 1.1.2.1. Model 2

| Test | Measure | Cohort 1 | Cohort 2 | Cohort 3 | All |
|---|---|---|---|---|---|
| TS POC T | Specificity | 98.5 [97 - 99.6] | 99.3 [98.8 - 99.8] | 99.4 [98.5 - 99.9] | 99.2 [98.8 - 99.6] |
| TS POC T | Sensitivity | 48.4 [3.3 - 96.9] | 40.3 [1 - 96] | 39.5 [1.1 - 95.6] | 44.4 [1.9 - 96.7] |
| TS POC T | PPV | 22.9 [0.5 - 69.6] | 11 [0.1 - 49.6] | 20.3 [0.3 - 74.1] | 10.2 [0.2 - 40.2] |
| TS POC T | NPV | 99.4 [97 - 100] | 99.8 [98.4 - 100] | 99.6 [97.5 - 100] | 99.8 [98.9 - 100] |
| TS POC CC | Specificity | 81.7 [74.3 - 87.5] | 97.3 [96.1 - 98.3] | 96.6 [94.3 - 98.3] | 94.4 [93 - 95.6] |
| TS POC CC | Sensitivity | 52.6 [9.4 - 92.6] | 43 [4.2 - 90.2] | 45.1 [5.3 - 90.7] | 48.2 [6.3 - 92.1] |
| TS POC CC | PPV | 2.8 [0.1 - 9.6] | 3.6 [0.1 - 14.7] | 6.1 [0.1 - 26] | 1.9 [0 - 7.2] |
| TS POC CC | NPV | 99.3 [96.9 - 100] | 99.8 [98.6 - 100] | 99.6 [97.9 - 100] | 99.8 [98.9 - 100] |
| rES33-EITB | Specificity | 93.1 [85.7 - 96.9] | 94.7 [89.3 - 98.1] | 96.9 [90 - 99.6] | 96.2 [93.2 - 98.2] |
| rES33-EITB | Sensitivity | 51.3 [15.7 - 86] | 48.9 [12.1 - 86.4] | 47 [11.5 - 84.8] | 49.6 [13.2 - 86] |
| rES33-EITB | PPV | 7.7 [0.3 - 27.7] | 3.1 [0 - 16.8] | 10.3 [0.1 - 49.2] | 3.5 [0.1 - 16.8] |
| rES33-EITB | NPV | 99.4 [97.7 - 100] | 99.8 [98.9 - 100] | 99.7 [98.2 - 100] | 99.8 [99.2 - 100] |
| copro-mPCR | Specificity | 96.2 [92 - 98.5] | 96.9 [93.2 - 98.7] | 96.5 [91.6 - 98.7] | 97.9 [95.7 - 98.8] |
| copro-mPCR | Sensitivity | 47.1 [18.7 - 77.8] | 47.2 [16.6 - 79.3] | 47.3 [17 - 79] | 46.6 [16.6 - 77.9] |
| copro-mPCR | PPV | 12.1 [0.5 - 40.3] | 4.8 [0.1 - 24.6] | 7.7 [0.1 - 33.6] | 5.3 [0.2 - 23.4] |
| copro-mPCR | NPV | 99.4 [97.7 - 100] | 99.8 [99 - 100] | 99.7 [98.3 - 100] | 99.8 [99.2 - 100] |
| copro-Ag | Specificity | 94.3 [87.4 - 97.9] | 93.4 [87.9 - 97.2] | 93 [84.7 - 97.8] | 95.3 [91.9 - 97.7] |
| copro-Ag | Sensitivity | 49.7 [25.3 - 74.6] | 48.1 [22 - 74.5] | 48.9 [23.4 - 74.6] | 49 [23.2 - 74.7] |
| copro-Ag | PPV | 9.2 [0.4 - 32.3] | 2.4 [0 - 13.3] | 4.4 [0.1 - 21.5] | 2.8 [0.1 - 13.4] |
| copro-Ag | NPV | 99.4 [97.8 - 100] | 99.8 [99 - 100] | 99.7 [98.4 - 100] | 99.8 [99.3 - 100] |
| Prevalence | | 1 [0 - 3.6] | 0.3 [0 - 1.7] | 0.6 [0 - 2.7] | 0.3 [0 - 1.3] |

### 1.1.2.1. Model 2B

| Test | Measure | Cohort 1 | Cohort 2 | Cohort 3 | All |
|---|---|---|---|---|---|
| TS POC T | Specificity | 98.4 [96.9 - 99.3] | 99.3 [98.7 - 99.7] | 99.3 [98.5 - 99.8] | 99.2 [98.8 - 99.5] |
| TS POC T | Sensitivity | 46.9 [2.9 - 96.8] | 39.9 [1.1 - 95.9] | 40.8 [1.2 - 96.2] | 44.7 [2 - 96.7] |
| TS POC T | PPV | 13.9 [0.3 - 44] | 6.9 [0.1 - 28.9] | 15 [0.2 - 56.6] | 5.8 [0.1 - 20.6] |
| TS POC T | NPV | 99.6 [97.7 - 100] | 99.8 [99 - 100] | 99.7 [98.3 - 100] | 99.9 [99.4 - 100] |
| TS POC CC | Specificity | 81.5 [74 - 87.3] | 97.3 [96 - 98.2] | 96.5 [94.2 - 98.1] | 94.3 [93 - 95.5] |
| TS POC CC | Sensitivity | 50.5 [8.1 - 92.3] | 42.7 [4.2 - 89.7] | 44.3 [5.1 - 90.4] | 47.5 [6.1 - 91.5] |
| TS POC CC | PPV | 1.6 [0.1 - 5.4] | 2.2 [0 - 8.9] | 3.8 [0.1 - 15.4] | 1 [0 - 3.4] |
| TS POC CC | NPV | 99.5 [97.4 - 100] | 99.9 [99.1 - 100] | 99.7 [98.4 - 100] | 99.9 [99.4 - 100] |
| rES33-EITB | Specificity | 92.9 [85.5 - 96.7] | 94.6 [89.2 - 98.1] | 96.9 [90.1 - 99.6] | 96.2 [93.2 - 98.2] |
| rES33-EITB | Sensitivity | 52.3 [15.7 - 86.8] | 49.4 [12.7 - 86.4] | 49.4 [12.7 - 86.2] | 51.4 [14.4 - 86.7] |
| rES33-EITB | PPV | 5.2 [0.2 - 20.5] | 2 [0 - 11.1] | 8.2 [0.1 - 42.1] | 2.1 [0 - 9.5] |
| rES33-EITB | NPV | 99.6 [98.4 - 100] | 99.9 [99.4 - 100] | 99.8 [98.9 - 100] | 99.9 [99.6 - 100] |
| copro-mPCR | Specificity | 96.2 [92 - 98.5] | 96.9 [93.1 - 98.7] | 96.5 [91.6 - 98.7] | 97.9 [95.7 - 98.8] |
| copro-mPCR | Sensitivity | 84.8 [75.8 - 93.9] | 84.8 [75.7 - 94.1] | 84.8 [75.7 - 94.1] | 84.8 [75.7 - 94] |
| copro-mPCR | PPV | 14.1 [0.6 - 45.9] | 5.7 [0.1 - 28.2] | 9.3 [0.2 - 39.4] | 5.9 [0.2 - 24.6] |
| copro-mPCR | NPV | 99.9 [99.5 - 100] | 100 [99.8 - 100] | 99.9 [99.7 - 100] | 100 [99.9 - 100] |
| copro-Ag | Specificity | 94.2 [87.3 - 97.9] | 93.4 [87.9 - 97.2] | 92.9 [84.5 - 97.8] | 95.3 [91.9 - 97.7] |
| copro-Ag | Sensitivity | 52.5 [25.9 - 77.6] | 50.2 [23.1 - 77.3] | 49.9 [22.9 - 77] | 52.2 [25.4 - 78] |
| copro-Ag | PPV | 6.7 [0.2 - 25.9] | 1.7 [0 - 9.3] | 3.2 [0 - 16] | 1.8 [0 - 8.3] |
| copro-Ag | NPV | 99.6 [98.6 - 100] | 99.9 [99.4 - 100] | 99.8 [99 - 100] | 99.9 [99.7 - 100] |
| Prevalence | | 0.7 [0 - 2.7] | 0.2 [0 - 1.1] | 0.4 [0 - 1.8] | 0.2 [0 - 0.7] |

### 1.1.2.1. Model 3

| Test | Measure | Cohort 1 | Cohort 2 | Cohort 3 | All |
|---|---|---|---|---|---|
| TS POC T | Specificity | 98.4 [97 - 99.4] | 99.3 [98.7 - 99.7] | 99.3 [98.5 - 99.8] | 99.2 [98.8 - 99.5] |
| TS POC T | Sensitivity | 45.3 [4.8 - 95.3] | 36.6 [0.8 - 95.3] | 40 [1.1 - 96] | 39.2 [2.5 - 94.1] |
| TS POC T | PPV | 18.8 [1.4 - 50.7] | 7 [0.1 - 28.8] | 14.6 [0.2 - 55.8] | 8.2 [0.5 - 24.3] |
| TS POC T | NPV | 99.4 [97.2 - 100] | 99.8 [98.5 - 100] | 99.7 [98.2 - 100] | 99.8 [98.9 - 100] |
| TS POC CC | Specificity | 81.7 [74.3 - 87.4] | 97.3 [96 - 98.2] | 96.5 [94.2 - 98.2] | 94.4 [93 - 95.5] |
| TS POC CC | Sensitivity | 54.4 [11.7 - 93.1] | 39.7 [2.9 - 88.9] | 44.3 [4.9 - 90.5] | 47.1 [6.2 - 91.3] |
| TS POC CC | PPV | 2.5 [0.2 - 6.9] | 2.3 [0 - 9.2] | 3.9 [0.1 - 15.8] | 1.6 [0.1 - 4.5] |
| TS POC CC | NPV | 99.4 [97 - 100] | 99.8 [98.5 - 100] | 99.7 [98.3 - 100] | 99.8 [98.9 - 100] |
| rES33-EITB | Specificity | 93 [85.6 - 96.8] | 94.6 [89.2 - 98.1] | 96.9 [90 - 99.6] | 96.2 [93.2 - 98.2] |
| rES33-EITB | Sensitivity | 50.3 [16.2 - 84.6] | 48 [11.3 - 86] | 49.3 [12.7 - 86.5] | 48.2 [12.8 - 84.2] |
| rES33-EITB | PPV | 6.8 [0.5 - 23.9] | 2.4 [0 - 13.8] | 8.2 [0.1 - 42.7] | 3.3 [0.2 - 14.2] |
| rES33-EITB | NPV | 99.5 [98 - 100] | 99.8 [98.9 - 100] | 99.8 [98.9 - 100] | 99.8 [99.2 - 100] |
| copro-mPCR | Specificity | 96.3 [92.1 - 98.5] | 97 [93.3 - 98.7] | 96.5 [91.6 - 98.7] | 97.9 [95.8 - 98.8] |
| copro-mPCR | Sensitivity | 84.5 [75.9 - 93.5] | 84.9 [75.4 - 94.4] | 84.8 [75.6 - 94.1] | 84.8 [75.7 - 94] |
| copro-mPCR | PPV | 18.8 [1.9 - 51.7] | 7.4 [0.1 - 38.6] | 9.5 [0.2 - 40.8] | 10.1 [0.7 - 38.4] |
| copro-mPCR | NPV | 99.8 [99.4 - 100] | 100 [99.7 - 100] | 99.9 [99.7 - 100] | 100 [99.8 - 100] |
| copro-Ag | Specificity | 96.4 [92 - 98.9] | 95.2 [91.2 - 98.2] | 95.5 [91 - 98.9] | 96.2 [93 - 98.4] |
| copro-Ag | Sensitivity | 54.2 [28.5 - 78.2] | 51 [23.4 - 78.6] | 50 [23.1 - 76.9] | 54.6 [27.9 - 79.5] |
| copro-Ag | PPV | 14.2 [1.2 - 44.2] | 3.2 [0 - 19.4] | 5 [0.1 - 25.6] | 4.2 [0.2 - 19.3] |
| copro-Ag | NPV | 99.5 [98.3 - 100] | 99.9 [99.2 - 100] | 99.8 [98.9 - 100] | 99.9 [99.4 - 100] |
| Prevalence | | 1 [0.1 - 3.2] | 0.3 [0 - 1.6] | 0.4 [0 - 2] | 0.3 [0 - 1.2] |

## 1.1.3. Without rES33-EITB

Priors:

| Model | Test | LL | UL |
|---|---|---|---|
| **Model 1** | Prevalence | 0.001 | 0.05 |
| | All - sensitivity | 0.000 | 1.00 |
| | All - specificity | 0.500 | 1.00 |
| **Model 2** | Prevalence | 0.001 | 0.05 |
| | mPCR-sensitivity | 0.700 | 0.99 |
| | mPCR-specificity | 0.900 | 0.99 |
| | Other - sensitivity | 0.000 | 1.00 |
| | Other - specificity | 0.500 | 1.00 |

Overview of the different models:

| Model | Variable | Cohort 1 | Cohort 2 | Cohort 3 | All |
|---|---|---|---|---|---|
| Model1 | Bayesian P | 0.705 | 0.790 | 0.550 | 0.771 |
| Model1 | deviance | 38.0 | 35.2 | 26.8 | 49.1 |
| Model2 | Bayesian P | 0.650 | 0.714 | 0.477 | 0.706 |
| Model2 | deviance | 36.4 | 32.7 | 25.0 | 47.3 |

### 1.1.3.1. Model 1

| Test | Measure | Cohort 1 | Cohort 2 | Cohort 3 | All |
|---|---|---|---|---|---|
| TS POC T | Specificity | 98.6 [97.1 - 99.7] | 99.4 [98.8 - 99.9] | 99.4 [98.6 - 99.9] | 99.3 [98.8 - 99.9] |
| TS POC T | Sensitivity | 43.2 [2 - 95.7] | 40.1 [0.9 - 96.2] | 37.8 [1.1 - 95.3] | 38.1 [1.1 - 94.8] |
| TS POC T | PPV | 25.7 [0.4 - 82.9] | 17.5 [0.2 - 79.4] | 26.2 [0.4 - 86.2] | 18.8 [0.2 - 83.9] |
| TS POC T | NPV | 99.2 [96.5 - 100] | 99.6 [97.2 - 100] | 99.4 [96.6 - 100] | 99.6 [97.6 - 100] |
| TS POC CC | Specificity | 81.9 [74.4 - 87.6] | 97.4 [96.1 - 98.5] | 96.7 [94.3 - 98.7] | 94.5 [93.1 - 95.7] |
| TS POC CC | Sensitivity | 52.5 [9.6 - 92.5] | 39.6 [3.3 - 89.1] | 45 [5.6 - 90.8] | 45.2 [5.4 - 91.2] |
| TS POC CC | PPV | 3.5 [0.1 - 11.9] | 5.5 [0.1 - 27] | 9.6 [0.1 - 52.7] | 3.6 [0.1 - 13.6] |
| TS POC CC | NPV | 99.2 [96.6 - 100] | 99.6 [97.6 - 100] | 99.5 [97.3 - 100] | 99.6 [97.8 - 100] |
| rES33-EITB | Specificity | 96.6 [89.3 - 99.5] | 97.6 [93.4 - 99.7] | 97.1 [90.3 - 99.7] | 98.7 [96.4 - 99.8] |
| rES33-EITB | Sensitivity | 43.8 [11.8 - 81.8] | 44.2 [9.2 - 84.7] | 43.2 [9.3 - 83.1] | 41.6 [8.8 - 82.7] |
| rES33-EITB | PPV | 17.9 [0.5 - 62.8] | 10.2 [0.1 - 60.5] | 13.8 [0.2 - 61] | 16.5 [0.4 - 71.5] |
| rES33-EITB | NPV | 99.2 [97.1 - 100] | 99.7 [97.9 - 100] | 99.5 [97.2 - 100] | 99.6 [98.1 - 100] |
| copro-mPCR | Specificity | 95.2 [88.1 - 98.7] | 94.4 [89 - 98.1] | 93.7 [85.1 - 98.5] | 96.1 [92.7 - 98.4] |
| copro-mPCR | Sensitivity | 48.9 [19.8 - 79.1] | 46.2 [14.8 - 79.9] | 46.9 [17.1 - 78.8] | 47.6 [16.2 - 80.7] |
| copro-mPCR | PPV | 13.4 [0.4 - 45.4] | 4.5 [0.1 - 26.8] | 6.8 [0.1 - 32.7] | 6.8 [0.1 - 33.3] |
| copro-mPCR | NPV | 99.3 [97.4 - 100] | 99.7 [98.1 - 100] | 99.5 [97.5 - 100] | 99.7 [98.4 - 100] |
| Prevalence | | 1.3 [0 - 4.1] | 0.5 [0 - 3] | 0.8 [0 - 3.7] | 0.6 [0 - 2.7] |

### 1.1.4. Model 2

| Test | Measure | Cohort 1 | Cohort 2 | Cohort 3 | All |
|---|---|---|---|---|---|
| TS POC T | Specificity | 98.4 [96.9 - 99.3] | 99.3 [98.7 - 99.7] | 99.3 [98.5 - 99.8] | 99.2 [98.8 - 99.5] |
| TS POC T | Sensitivity | 43.3 [2.8 - 95.5] | 37.7 [0.8 - 95.6] | 40.8 [1.2 - 95.9] | 37.4 [1.4 - 94] |
| TS POC T | PPV | 15 [0.4 - 45.5] | 7.2 [0.1 - 29.7] | 15.4 [0.2 - 58.6] | 6.7 [0.2 - 22.2] |
| TS POC T | NPV | 99.4 [97.4 - 100] | 99.8 [98.6 - 100] | 99.7 [98.2 - 100] | 99.8 [98.9 - 100] |
| TS POC CC | Specificity | 81.6 [74.1 - 87.4] | 97.3 [96 - 98.2] | 96.5 [94.2 - 98.2] | 94.3 [93 - 95.5] |
| TS POC CC | Sensitivity | 51.9 [9.3 - 92.4] | 40.3 [3.2 - 89.3] | 44 [4.7 - 90.2] | 45.8 [5.5 - 90.8] |
| TS POC CC | PPV | 2 [0.1 - 6.2] | 2.3 [0 - 9.2] | 3.9 [0.1 - 16] | 1.4 [0.1 - 4.2] |
| TS POC CC | NPV | 99.4 [97.1 - 100] | 99.8 [98.6 - 100] | 99.7 [98.3 - 100] | 99.8 [98.9 - 100] |
| rES33-EITB | Specificity | 97.3 [92.5 - 99.7] | 97.7 [93.6 - 99.7] | 97.5 [92 - 99.8] | 98.7 [96.4 - 99.8] |
| rES33-EITB | Sensitivity | 84.6 [74 - 95.5] | 84.8 [73.7 - 96.2] | 84.7 [73.8 - 95.7] | 84.7 [73.8 - 95.9] |
| rES33-EITB | PPV | 24.5 [1.3 - 74.5] | 11.2 [0.1 - 63.6] | 16.7 [0.3 - 70.5] | 17.2 [0.5 - 72.6] |
| rES33-EITB | NPV | 99.9 [99.5 - 100] | 100 [99.8 - 100] | 99.9 [99.7 - 100] | 100 [99.8 - 100] |
| copro-mPCR | Specificity | 95.3 [88.2 - 98.6] | 94.4 [89 - 98] | 93.7 [85.1 - 98.4] | 96 [92.6 - 98.3] |
| copro-mPCR | Sensitivity | 55.1 [22.8 - 83.7] | 51.1 [18.1 - 83.9] | 49.8 [17.6 - 82] | 55.9 [22.8 - 85.3] |
| copro-mPCR | PPV | 10.2 [0.4 - 37] | 2.7 [0 - 17] | 3.9 [0.1 - 20.6] | 3.7 [0.1 - 18.5] |
| copro-mPCR | NPV | 99.6 [98.4 - 100] | 99.9 [99.3 - 100] | 99.8 [98.9 - 100] | 99.9 [99.5 - 100] |
| Prevalence | | 0.8 [0 - 3] | 0.3 [0 - 1.5] | 0.4 [0 - 2] | 0.2 [0 - 1.1] |

## 1.2. Round 2 (new priors)

### 1.2.1. Priors - rationale

The new priors were based on the output of Model 1 of Mubanga et al. [1]. The priors were wider (the LL was rounded down and UL rounded up) than the 95% CI since the diagnostic performance was evaluated in different populations (community vs. hospitals). The upper level of the prior for the sensitivity of copro Ag ELISA was further increased based on results of Praet et al. [2]. The lower level for the specificity of copro Ag ELISA was decreased to account for potential cross-reacting diseases, which may be more likely in hospital patients than in healthy community members. The same priors were used for the three cohorts, since there was no prior knowledge to adapt the priors according to the cohort.

| | **Rationale: 95% CI of Model 1 in Mubanga et al** | | **New priors that were used in the current paper** | |
|---|---|---|---|---|
| **Test** | LL | UL | LL | UL |
| **TS POC-T – sensitivity** | 0.01 | 0.95 | 0.01 | 0.95 |
| **TS POC-T – specificity** | 0.98 | 1.00 | 0.95 | 1.00 |
| **mPCR – sensitivity** | 0.11 | 0.82 | 0.10 | 0.85 |
| **mPCR – specificity** | 0.95 | 1.00 | 0.95 | 1.00 |
| **rES33-EITB – sensitivity** | 0.10 | 0.94 | 0.10 | 0.95 |
| **rES33-EITB – specificity** | 0.64 | 0.78 | 0.50 | 0.99 |
| **Copro Ag ELISA – sensitivity** | 0.17 | 0.78 | 0.15 | 0.99 |
| **Copro Ag ELISA – specificity** | 0.85 | 0.94 | 0.30 | 0.99 |
| **Prevalence** | | | 0.00 | 0.05 |

## 1.2.2. Model output - including rES33-EITB

Diagnostic accuracy measures:

| Test | Measure | Cohort 1* | Cohort 2* | Cohort 3* | All |
|---|---|---|---|---|---|
| TS POC T | Sensitivity | 50.2 [4.9 - 96.4] | 40.8 [2.2 - 95.2] | 40.4 [2.3 – 95.0] | 44.4 [2.9 - 95.5] |
| | Specificity | 98.6 [97.1 - 99.6] | 99.3 [98.7 - 99.7] | 99.4 [98.5 - 99.9] | 99.2 [98.8 - 99.6] |
| | PPV | 25 [0.9 - 71.6] | 10 [0.2 - 41.2] | 20.1 [0.4 - 73.1] | 10.2 [0.3 - 35.3] |
| | NPV | 99.4 [97.2 - 100] | 99.8 [98.8 - 100] | 99.6 [97.9 - 100] | 99.8 [98.9 - 100] |
| TS POC CC | Sensitivity | 53.7 [10.8 - 92.6] | 43.3 [4.8 - 90] | 44.9 [5.6 - 90.5] | 48.9 [6.7 - 91.9] |
| | Specificity | 81.8 [74.3 - 87.5] | 97.3 [96 - 98.3] | 96.6 [94.3 - 98.2] | 94.4 [93 - 95.5] |
| | PPV | 2.9 [0.1 - 9] | 3.1 [0.1 - 12.5] | 5.4 [0.1 - 21.7] | 1.8 [0.1 - 6.3] |
| | NPV | 99.3 [96.9 - 100] | 99.8 [98.8 - 100] | 99.7 [98 - 100] | 99.8 [99 - 100] |
| rES33-EITB | Sensitivity | 53.5 [23.4 - 82.9] | 51.8 [20.9 - 83.2] | 50.7 [20.7 - 82.3] | 52 [21.4 - 82.6] |
| | Specificity | 92.3 [85.1 - 96] | 94.6 [89.2 - 98] | 95.9 [89.1 - 98.6] | 96.1 [93.1 - 97.9] |
| | PPV | 7.3 [0.4 - 24.1] | 2.8 [0 - 14.7] | 6.9 [0.1 - 31.1] | 3.5 [0.1 - 15] |
| | NPV | 99.4 [97.9 - 100] | 99.9 [99.2 - 100] | 99.7 [98.7 - 100] | 99.9 [99.3 - 100] |
| copro-mPCR | Sensitivity | 46.3 [25.3 - 68.1] | 46.4 [24.4 - 69] | 46.4 [24.6 - 69] | 46.5 [24.8 - 68.7] |
| | Specificity | 98 [95.8 - 99.6] | 97.9 [95.5 - 99.6] | 98.2 [95.5 - 99.8] | 98.6 [96.4 - 99.7] |
| | PPV | 21.7 [1.3 - 64.1] | 6.9 [0.1 - 37.9] | 14.6 [0.3 - 62.8] | 10 [0.3 - 46.7] |
| | NPV | 99.4 [97.9 - 100] | 99.8 [99.2 - 100] | 99.7 [98.6 - 100] | 99.8 [99.3 - 100] |
| copro-Ag | Sensitivity | 56.5 [37.8 - 75.4] | 56.1 [36.7 - 75.9] | 56.3 [37 - 75.9] | 56.6 [37.5 - 76] |
| | Specificity | 93.1 [86 - 96.9] | 93.1 [87.5 - 97] | 92.2 [83.7 - 97.1] | 95.1 [91.6 - 97.6] |
| | PPV | 8.7 [0.5 - 28.2] | 2.4 [0 - 12.4] | 4.1 [0.1 - 18.9] | 3.2 [0.1 - 14.4] |
| | NPV | 99.5 [98.2 - 100] | 99.9 [99.3 - 100] | 99.8 [98.8 - 100] | 99.9 [99.4 - 100] |
| Prevalence | | 1.1 [0.1 - 3.5] | 0.3 [0 - 1.4] | 0.5 [0 - 2.4] | 0.3 [0 - 1.2] |

*: used as final model

| Variable | Cohort 1* | Cohort 2* | Cohort 3* | All |
|---|---|---|---|---|
| Bayesian P | 0.631 | 0.596 | 0.424 | 0.662 |
| deviance | 54.5 | 47.7 | 32.7 | 70.6 |

### 1.2.3. Model output - without rES33-EITB

Diagnostic accuracy measures:

| Test | Measure | Cohort 1 | Cohort 2 | Cohort 3 | All |
|---|---|---|---|---|---|
| TS POC T | Specificity | 98.6 [97.1 - 99.6] | 99.3 [98.8 - 99.7] | 99.4 [98.5 - 99.9] | 99.2 [98.8 - 99.6] |
| | Sensitivity | 44.1 [4 - 91.5] | 38.4 [2 - 91.3] | 37.7 [2.1 - 91] | 36.7 [2.2 - 90.3] |
| | PPV | 26 [1.1 - 75.8] | 10.9 [0.2 - 48.3] | 22.7 [0.4 - 79] | 11.5 [0.4 - 40.7] |
| | NPV | 99.2 [96.7 - 100] | 99.7 [98.3 - 100] | 99.5 [97.4 - 100] | 99.7 [98.2 - 100] |
| TS POC CC | Specificity | 81.8 [74.4 - 87.6] | 97.3 [96 - 98.3] | 96.6 [94.3 - 98.3] | 94.4 [93 - 95.6] |
| | Sensitivity | 54.2 [11.6 - 92.4] | 41.1 [4 - 88.8] | 44.5 [5.6 - 90] | 47.6 [6.3 - 91.5] |
| | PPV | 3.5 [0.2 - 10.8] | 3.2 [0.1 - 13.1] | 6.7 [0.1 - 28.1] | 2.5 [0.1 - 8.7] |
| | NPV | 99.2 [96.6 - 100] | 99.8 [98.3 - 100] | 99.6 [97.7 - 100] | 99.7 [98.3 - 100] |
| copro-mPCR | Specificity | 98.1 [95.8 - 99.7] | 98 [95.5 - 99.7] | 98.2 [95.4 - 99.9] | 98.7 [96.5 - 99.8] |
| | Sensitivity | 45.1 [20 - 72.4] | 45.9 [19 - 74.2] | 44.7 [18.7 - 73.2] | 45.1 [19 - 73.4] |
| | PPV | 25.8 [1.7 - 74.2] | 8.8 [0.1 - 53.8] | 17.3 [0.3 - 71.2] | 15.5 [0.5 - 70] |
| | NPV | 99.2 [97.4 - 100] | 99.8 [98.9 - 100] | 99.6 [98.2 - 100] | 99.8 [98.8 - 100] |
| copro-Ag | Specificity | 94.5 [87.3 - 97.8] | 94.2 [88.7 - 97.8] | 93.4 [84.7 - 97.9] | 95.8 [92.4 - 98.1] |
| | Sensitivity | 56.5 [33.3 - 80.1] | 55.7 [31.5 - 80.9] | 55.6 [31.8 - 80.4] | 57.2 [32.9 - 81.6] |
| | PPV | 12.7 [0.8 - 38.7] | 3.4 [0 - 19.7] | 5.9 [0.1 - 27.1] | 5.8 [0.2 - 27.5] |
| | NPV | 99.4 [97.9 - 100] | 99.8 [99.2 - 100] | 99.7 [98.5 - 100] | 99.8 [99.1 - 100] |
| Prevalence | | 1.3 [0.1 - 4] | 0.3 [0 - 1.9] | 0.6 [0 - 2.9] | 0.4 [0 - 1.9] |

| Variable | Cohort 1 | Cohort 2 | Cohort 3 | All |
|---|---|---|---|---|
| Bayesian P | 0.635 | 0.681 | 0.443 | 0.642 |
| deviance | 35.9 | 31.9 | 24.2 | 45.9 |

## 1.3. Directed acyclic graph taeniosis

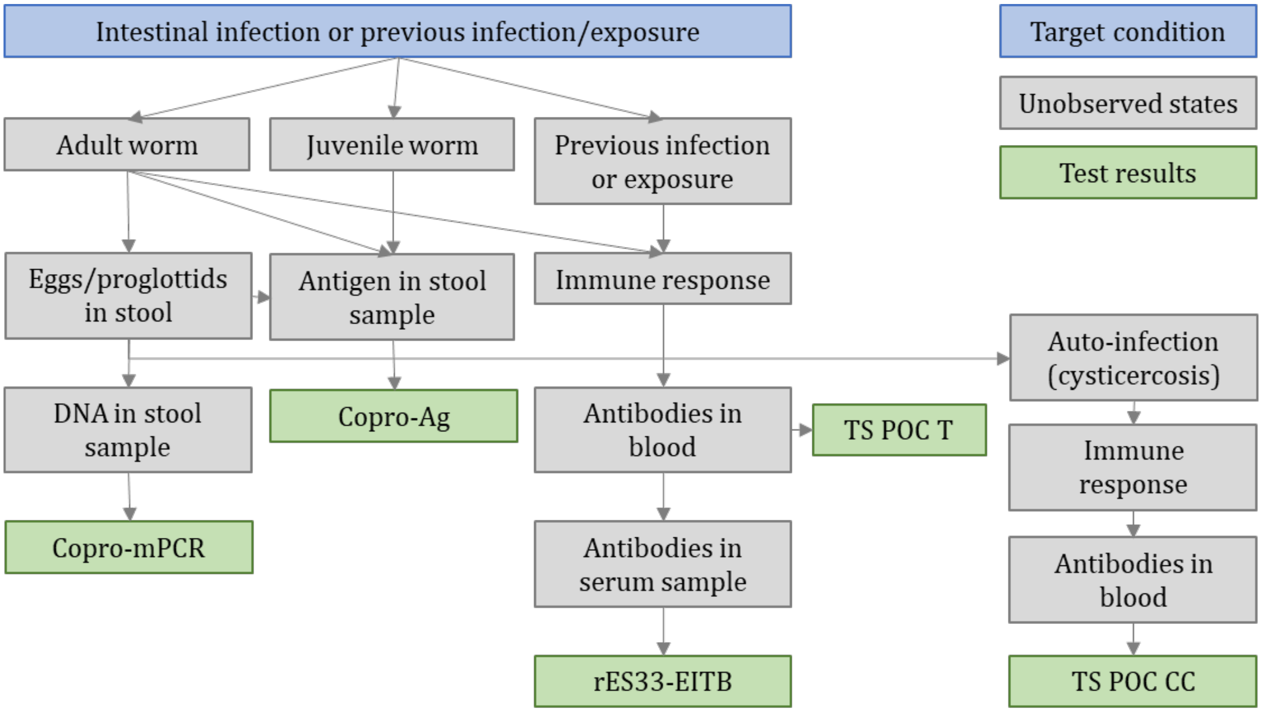


# 2. Cysticercosis

Initially the same priors were used as the least restrictive model in the community study [3]. The priors and output of these models are described in section 2.1. However, as the current study used a different study population and test performance is known to differ according to the population, new priors were defined, which are described in section 2.2. For patients in cohort 2, model 1 using the original priors was used as final model. For cohort 1 and cohort 3, the models using the new priors were used. The models marked with an asterisk (*) have been selected as the definitive models for the manuscript.

## 2.1. Round 1 (original priors)

Priors taken from Mubanga et al. [3]:

| Model | Test | LL | UL |
|---|---|---|---|
| **Model 1** | Prevalence | 0.05 | 0.30 |
| | All - sensitivity | 0.00 | 1.00 |
| | All - specificity | 0.50 | 1.00 |
| **Model 2** | Prevalence | 0.05 | 0.30 |
| | serumAg-sensitivity | 0.50 | 0.99 |
| | serumAg-specificity | 0.90 | 0.99 |
| | Other sensitivity | 0.00 | 1.00 |
| | Other specificity | 0.50 | 1.00 |
| **Model 3** | Prevalence | 0.05 | 0.30 |
| | serumAg-sensitivity | 0.50 | 0.99 |
| | serumAg-specificity | 0.90 | 0.99 |
| | rT24 - sensitivity | 0.90 | 0.99 |
| | rT24 - specificity | 0.90 | 0.99 |
| | Other sensitivity | 0.00 | 1.00 |
| | Other specificity | 0.50 | 1.00 |

| Variable | Model | Cohort 1 | Cohort 2 | Cohort 3 |
|---|---|---|---|---|
| Bayesian P | Model 1 | 0.630 | 0.635* | 0.665 |
| deviance | Model 1 | 27.9 | 28.2* | 24.3 |
| Bayesian P | Model 2 | 0.597 | 0.636 | 0.630 |
| deviance | Model 2 | 27.4 | 28.2 | 23.8 |
| Bayesian P | Model 3 | 0.570 | 0.804 | 0.882 |
| deviance | Model 3 | 27.1 | 30.7 | 28.2 |

### 2.1.1. Model 1

| Test | Measure | Cohort 1 | Cohort 2* | Cohort 3 |
|---|---|---|---|---|
| TS POC CC | Sensitivity | 77.7 [40.3 - 99.2] | 24.9 [6.4 - 52.7] | 17.9 [0.8 - 57.5] |
| TS POC CC | Specificity | 93.5 [86.5 - 99.5] | 99.1 [97.8 - 100] | 97.8 [95.1 - 99.8] |
| TS POC CC | PPV | 68.8 [42 - 97.6] | 71.6 [35.4 - 98.8] | 42.1 [2.6 - 94.8] |
| TS POC CC | NPV | 95.1 [82.4 - 99.9] | 92.4 [77.4 - 97.3] | 91.2 [75.6 - 97.4] |
| serumAg ELISA | Sensitivity | 65.5 [37.8 - 93.7] | 42.7 [9.5 - 86.2] | 48.1 [6.6 - 92.6] |
| serumAg ELISA | Specificity | 94.1 [84.8 - 99.1] | 95.9 [89.9 - 99.6] | 91.7 [80.2 - 98.9] |
| serumAg ELISA | PPV | 68.7 [39.2 - 93.9] | 50.9 [15.1 - 94.6] | 39.8 [5.8 - 89] |
| serumAg ELISA | NPV | 92.8 [81.4 - 99.3] | 93.8 [78.4 - 99] | 93.8 [77.8 - 99.3] |
| rT24H-EITB | Sensitivity | 64 [38.1 - 90.5] | 40.7 [12 - 77] | 33.5 [6.6 - 73.9] |
| rT24H-EITB | Specificity | 95.1 [87.9 - 98.9] | 96 [90.6 - 99.3] | 95.6 [88.6 - 99.2] |
| rT24H-EITB | PPV | 71.9 [45.3 - 92.4] | 50 [18.6 - 88.7] | 45.1 [11.5 - 84.1] |
| rT24H-EITB | NPV | 92.6 [81.6 - 99] | 93.6 [78.8 - 98.4] | 92.5 [77.3 - 98.1] |
| Prevalence | | 16.2 [8.2 - 27.6] | 9.4 [5.1 - 23.9] | 10 [5.1 - 25] |

### 2.1.2. Model 2

| Test | Measure | Cohort 1 | Cohort 2 | Cohort 3 |
|---|---|---|---|---|
| TS POC CC | Sensitivity | 80.5 [47.6 - 99.3] | 26.7 [10.1 - 52.2] | 21.4 [4.6 - 54.3] |
| TS POC CC | Specificity | 93 [87 - 99.2] | 99 [97.9 - 99.9] | 98 [96 - 99.6] |
| TS POC CC | PPV | 66.3 [45.2 - 96] | 66.3 [37.5 - 97.4] | 48.2 [16.4 - 89.6] |
| TS POC CC | NPV | 96.1 [86.4 - 99.9] | 94.3 [88.2 - 97.3] | 92.4 [81.3 - 97.2] |
| serumAg ELISA | se | 77.5 [55.7 - 96] | 70.6 [53.8 - 92.7] | 73.9 [53.7 - 95] |
| SensitivityrumAg ELISA | Specificity | 95.5 [90.8 - 98.6] | 96.5 [91.9 - 98.9] | 94.8 [90.4 - 98.7] |
| serumAg ELISA | PPV | 74.9 [52.7 - 92] | 62.6 [34.4 - 86.6] | 58.5 [31 - 88.9] |
| serumAg ELISA | NPV | 95.7 [88.6 - 99.5] | 97.6 [94.2 - 99.5] | 97.3 [91.7 - 99.6] |
| rT24H-EITB | Sensitivity | 68.8 [45.5 - 90.2] | 42.8 [18.2 - 75.2] | 36.2 [10 - 73.4] |
| rT24H-EITB | Specificity | 95.4 [88.5 - 98.9] | 95.6 [90.5 - 99] | 96.2 [89.6 - 99.3] |
| rT24H-EITB | PPV | 72.9 [46.8 - 92.3] | 45.1 [18.4 - 82.2] | 49.2 [15 - 86] |
| rT24H-EITB | NPV | 94.2 [85.5 - 98.9] | 95.4 [89.9 - 98.4] | 93.7 [83.8 - 98.1] |
| Prevalence | | 15 [8.2 - 25.1] | 7.3 [5.1 - 13.2] | 9 [5.1 - 19.7] |

### 2.1.3. Model 3

| Test | Measure | Cohort 1 | Cohort 2 | Cohort 3 |
|---|---|---|---|---|
| TS POC CC | Sensitivity | 83.6 [53.2 - 99.4] | 24.5 [11.8 - 42] | 17.2 [4 - 41.4] |
| TS POC CC | Specificity | 90.8 [86.3 - 94.2] | 98.6 [97.7 - 99.2] | 97.4 [95.4 - 98.8] |
| TS POC CC | PPV | 54.5 [43.1 - 65.7] | 52.1 [34.4 - 69.6] | 31.5 [10.2 - 57.9] |
| TS POC CC | NPV | 97.4 [90.5 - 99.9] | 95.2 [91.9 - 96.8] | 94 [88.2 - 96.6] |
| serumAg ELISA | Sensitivity | 87.7 [75.6 - 96.8] | 73.2 [57.5 - 94.4] | 75.1 [54.2 - 95.6] |
| serumAg ELISA | Specificity | 95.1 [90.8 - 98.4] | 95 [90.9 - 98.3] | 93.5 [90.2 - 97.7] |
| serumAg ELISA | PPV | 70.9 [50.2 - 89] | 50.5 [31.3 - 75.2] | 46.8 [28.5 - 73.6] |
| serumAg ELISA | NPV | 98.2 [95.6 - 99.6] | 98.2 [96.2 - 99.6] | 98 [95.3 - 99.7] |
| rT24H-EITB | Sensitivity | 94 [90.8 - 97.8] | 94.4 [91.4 - 97.5] | 94.3 [91.1 - 97.7] |
| rT24H-EITB | Specificity | 96.2 [91.6 - 98.5] | 96.4 [92.2 - 98.7] | 96.5 [91.7 - 98.7] |
| rT24H-EITB | PPV | 77 [55.8 - 90.8] | 64.5 [42 - 84] | 67.6 [42.5 - 86.5] |
| rT24H-EITB | NPV | 99.2 [98.4 - 99.8] | 99.6 [99.3 - 99.8] | 99.6 [99 - 99.8] |
| Prevalence | | 11.8 [7.4 - 18.6] | 6.2 [5 - 9.3] | 7 [5 - 12.4] |

## 2.2. Round 2 (new priors)

### 2.2.1. Priors - rationale

Priors were based on the output of the model using the least restrictive priors in Mubanga et al [3]. For patients in cohort 2 and cohort 3, we assumed that the CC disease spectrum was similar to the asymptomatic community members in Zambia, so the upper and lower limits of the priors were based on the limits of the 95% credible interval of model using the least restrictive priors. However, as the CC disease spectrum in cohort 1 (i.e. patients with signs/symptoms compatible with NCC) is expected to be different from the other two cohorts, different priors were used for cohort 1 than for the others. For cohort 1, the upper levels of sensitivity were increased to 100%, assuming that the sensitivities of diagnostic tests are higher in this group of more severely diseased patients.

| | | Rationale: results of the 95% CI of the least restrictive model in asymptomatic community members (taken from Mubanga et al. [3]) | | Priors used | | | |
|---|---|---|---|---|---|---|---|
| | | | | Cohort 1 | | Cohort 2 & 3 | |
| | | LL | UL | LL | UL | LL | UL |
| **Prevalence** | | 0.05 | 0.27 | 0.05 | 0.5 | 0.00 | 0.30 |
| **TS POC CC** | Sensitivity | 0.02 | 0.95 | 0.02 | 1.0 | 0.02 | 0.95 |
| **TS POC CC** | Specificity | 0.82 | 0.98 | 0.80 | 1.0 | 0.80 | 1.00 |
| **rT24H-EITB** | Sensitivity | 0.06 | 0.78 | 0.05 | 1.0 | 0.05 | 0.95 |
| **rT24H-EITB** | Specificity | 0.86 | 0.98 | 0.85 | 1.0 | 0.85 | 1.00 |
| **serum Ag ELISA** | Sensitivity | 0.11 | 0.75 | 0.10 | 1.0 | 0.10 | 0.80 |
| **serum Ag ELISA** | Specificity | 0.81 | 0.96 | 0.80 | 1.0 | 0.80 | 1.00 |

## 2.2.2. Model output

| **Test** | **Measure** | **Cohort 1*** | **Cohort 2** | **Cohort 3*** |
|---|---|---|---|---|
| TS POC CC | Sensitivity | 77.5 [37.8 - 99.2] | 48.2 [10.1 - 91.9] | 44.2 [6.6 - 91.5] |
| TS POC CC | Specificity | 92.3 [86.5 - 98.8] | 99.0 [98.0 - 99.9] | 98.1 [96.1 - 99.7] |
| TS POC CC | PPV | 63.3 [44.1 - 94.5] | 63.9 [37.6 - 96.4] | 46.6 [13.5 - 91.2] |
| TS POC CC | NPV | 95.1 [79.4 - 99.9] | 96.8 [86.2 - 99.9] | 96.4 [84.2 - 99.9] |
| serumAg ELISA | Sensitivity | 72.8 [41.3 - 95.7] | 57.0 [19.3 - 88.8] | 56.4 [19.0 - 88.5] |
| serumAg ELISA | Specificity | 94.9 [87.5 - 99.2] | 95.3 [89.8 - 99.2] | 92.2 [85.8 - 98.5] |
| serumAg ELISA | PPV | 72.2 [45.1 - 95.1] | 36.6 [11.9 - 86.4] | 27.1 [4.7 - 81.8] |
| serumAg ELISA | NPV | 94.5 [80.9 - 99.5] | 97.3 [87.9 - 99.8] | 97.3 [87.8 - 99.8] |
| rT24H-EITB | Sensitivity | 71.7 [44.5 - 92.5] | 50.7 [24.9 - 71.8] | 42.7 [20.2 - 66.7] |
| rT24H-EITB | Specificity | 96.2 [89.3 - 99.3] | 95.9 [90.8 - 99.2] | 96.7 [90.4 - 99.5] |
| rT24H-EITB | PPV | 77.3 [50.0 - 94.9] | 37.6 [11.8 - 84.3] | 40.9 [7.7 - 85.5] |
| rT24H-EITB | NPV | 94.4 [81.8 - 99.2] | 97.2 [88.7 - 99.5] | 96.7 [87.1 - 99.6] |
| Prevalence | | 15.3 [8.1 - 29.4] | 4.9 [1.3 - 15.2] | 5.1 [0.8 - 17.1] |

* used as final model

| **Variable** | **Cohort 1*** | **Cohort 2** | **Cohort 3*** |
|---|---|---|---|
| Bayesian P | 0.594 | 0.790 | 0.515 |
| deviance | 27.3 | 30.3 | 22.1 |

## 2.3. Directed acyclic graph cysticercosis

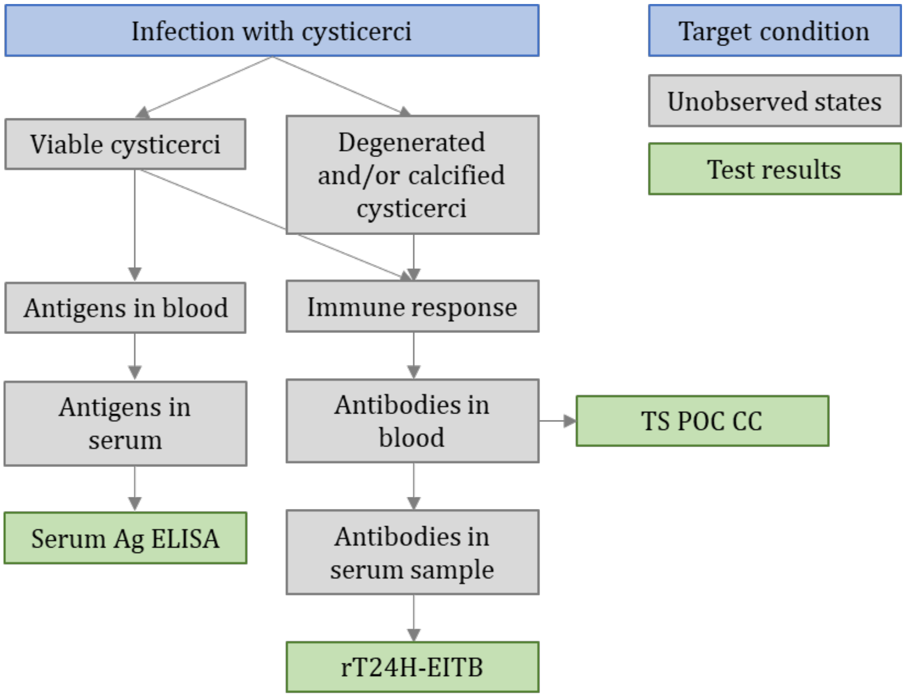

## 3. References

1. Mubanga C, Trevisan C, Van Damme I, Schmidt V, Phiri IK, Zulu G, et al. Challenges Encountered When Evaluating an Antibody-Detecting Point-of-Care Test for Taeniosis in an Endemic Community in Zambia: A Prospective Diagnostic Accuracy Study. Diagnostics. 2021;11: 2039. doi:10.3390/diagnostics11112039

2. Praet N, Verweij JJ, Mwape KE, Phiri IK, Muma JB, Zulu G, et al. Bayesian modelling to estimate the test characteristics of coprology, coproantigen ELISA and a novel real-time PCR for the diagnosis of taeniasis. Tropical Medicine & International Health. 2013;18: 608–614. doi:10.1111/tmi.12089

3. Mubanga C, Van Damme I, Trevisan C, Schmidt V, Phiri IK, Zulu G, et al. Evaluation of an Antibody Detecting Point of Care Test for Diagnosis of Taenia solium Cysticercosis in a Zambian Rural Community: A Prospective Diagnostic Accuracy Study. Diagnostics. 2021;11: 2121. doi:10.3390/diagnostics11112121
