# Supplementary material for: Evaluation of a rapid lateral flow assay for the detection of taeniosis and cysticercosis at district hospital level in Tanzania: A prospective multicentre diagnostic accuracy study
Source: PLoS Negl Trop Dis. 2025 Mar 28;19(3):e0012310. doi: 10.1371/journal.pntd.0012310 (PMC11977959; doi:10.1371/journal.pntd.0012310)
Supplement: S1 Checklist — (DOCX) [file pntd.0012310.s004.docx]

|  | **Section & Topic** | **No** | **Item** | **Reported in section #** |
| --- | --- | --- | --- | --- |
|  |  |  |  |  |
|  | **TITLE OR ABSTRACT** |  |  |  |
|  |  | **1** | Identification as a study of diagnostic accuracy, using at least one measure of accuracy (such as sensitivity, specificity, predictive values, or AUC) **and Bayesian latent class models** | The title includes “a prospective multicentre diagnostic accuracy study”. The abstract includes “Lacking a gold standard test for both infections, evaluating the diagnostic accuracy measures was done using the results of different coprological and serological tests in a Bayesian Latent Class Model approach.” |
|  | **ABSTRACT** |  |  |  |
|  |  | **2** | Structured summary of study design, methods, results, and conclusions  (for specific guidance, see STARD for Abstracts) | The abstract includes all required information. |
|  | **INTRODUCTION** |  |  |  |
|  |  | **3** | Scientific and clinical background, including the intended use and clinical role of the **tests under evaluation** | “The focus of this paper is the evaluation of the TS POC test for the detection of infection with the adult tapeworm (taeniosis) and/or the larval stage (cysticercosis) of *T. solium* in individuals attending district hospitals (Tier 2 level) in Tanzania.” |
|  |  | **4** | Study objectives and hypotheses, **such as estimation of diagnostic accuracy of the tests for a defined purpose through BLCM** | “The primary endpoints of this paper are the sensitivity and specificity of the TS POC test for the detection of both infections, evaluated in three different clinical cohorts.” |
|  | **METHODS** |  |  |  |
|  | *Study design* | **5** | Whether data collection was planned before the **tests** were performed (prospective study) or after (retrospective study) | Section 2.1 Study design: “The study was designed as a prospective, two-phase, multicentre diagnostic accuracy study. First, all participants were tested using the TS POC test (phase 1), after which all participants testing positive and a subset of the participants testing negative were requested to provide a blood and stool sample for further reference testing (phase 2).” |
|  | *Participants* | **6** | Eligibility criteria **and description of the source population** | Section 2.2 Participants: “To be included, individuals aged 10 years or above had to be willing and able to provide written informed consent, living in the study area for the past three months and planning to stay in the same area throughout the study period. Pregnant women and individuals with acute severe illness that needed in-patient care were excluded. Different additional criteria were used to be included in the different cohorts. ” |
|  |  | **7** | On what basis potentially eligible participants were identified  (such as symptoms, results from previous tests, inclusion in registry) | Section 2.2 Participants: “Individuals with signs/symptoms compatible with NCC (cohort 1) were consecutively recruited from Outpatient Departments (OPD) and the Mental Health Clinics (MHC). A questionnaire consisting of nine questions was used to assess if individuals fulfilled the criteria for epileptic seizures and/or severe progressive headache (S1 Appendix; more details can be found in Stelzle et al. [14,16]). For cohort 2, individuals who presented at the OPD with complaints compatible with intestinal worm infection were included. They needed to have at least one of the following signs/symptoms: abdominal pain/discomfort, having seen worm parts in the stool, express having a worm in the stomach, nausea, diarrhoea and loss of appetite. All other people presenting at the OPD, without signs/symptoms compatible with cohort 1 or cohort 2, were potential candidates to be included in cohort 3. Participants with epilepsy and/or headache (cohort 1) or symptoms compatible with intestinal worm infections (cohort 2) were consecutively recruited. For cohort 3, every 10^th^ individual was approached for enrolment in the study. ” |
|  |  | **8** | Where and when potentially eligible participants were identified (setting, location and dates) | Section 2.2 Participants: “Participants were enrolled between December 2017 and February 2020 in three district hospitals in southern Tanzania: Mbeya (rural) District (Ifisi) and Rungwe District (Tukuyu), both located in Mbeya Region, and Mbozi District (Vwawa), located in Songwe Region.” |
|  |  | **9** | Whether participants formed a consecutive, random or convenience series | Section 2.2 Participants: Patients were consecutively recruited. |
|  | *Test methods* | **10** | **Description of the tests under evaluation**, in sufficient detail to allow replication, **and/or cite references** | Section 2.3 and 2.4. “Details about the TS POC test and testing procedures have been described elsewhere [10,12,13,19]. In short, 20 µL blood was collected from a fingertip and placed in a sample port of one of the test strips, and the same procedure was repeated for the other test strip. After applying chase buffer, the TS POC test was read after 20 minutes. A TS POC test strip was considered positive when the control line and test line were positive (visible as a red line). The result was invalid when the control line was negative. The result was negative when the control line was positive and test line negative. The results were read by two different readers. When there was disagreement between the readers, the result of a third reader was decisive.”  “For taeniosis, the following three tests were used: 1) copro Ag ELISA according to Allan et al. [23] and modified by Mwape et al. [24] to detect *Taenia* antigens in stool using a predefined cut-off value of 0.972; 2) copro mPCR according to Yamasaki et al. [7] to detect *Taenia* DNA in stool, which was considered positive only when a 720 bp *T. solium* band was visible; and 3) rES33-EITB to detect antibodies in serum [25], which was positive when the rES33 band was visible. For cysticercosis, two serum-based tests were used: 1) serum Ag ELISA to detect antigens with the cut-off value determined by comparing the optical density to that of a set of negative serum samples according to Dorny *et al*. [26], and 2) rT24H-EITB to detect antibodies [20], which was considered positive when the rT24H band was visible. Although also the LLGP-EITB was initially planned to be included in the study [14], this in-house test could not be used due to doubts about the validity of the test results. The test did not satisfy internal quality control standards during laboratory analyses [16], so it was decided to exclude the results to ensure the reliability of the results presented in this study. Further details regarding the tests are available in the study protocols [13,14].” |
|  |  | **11** | Rationale for choosing the **tests under evaluation in relation to their purpose** | Section 2.3 and 2.4. “The TS POC is an antibody-detecting lateral flow assay, using two test strips, each with a previously characterised recombinant protein, rES33 for the TS POC T test strip and rT24H for the TS POC CC test strip [20,21]. During initial assessments conducted within laboratory settings using known positive and negative control sera, the TS POC test demonstrated encouraging results (unpublished data). The TS POC T test strip exhibited a sensitivity of 82% and a specificity of 99% in detecting taeniosis. In the case of NCC, the TS POC CC test strip had a sensitivity of 88%, and 93% for infection with multiple cysticerci, with a corresponding specificity of 99%.”  “For taeniosis, the following three tests were used: 1) copro Ag ELISA according to Allan et al. [20] and modified by Mwape et al. [21] to detect *Taenia* antigens in stool using a predefined cut-off value; 2) copro mPCR according to Yamasaki et al. [7] to detect *Taenia* DNA in stool, which was considered positive only when a 720 bp *T. solium* band was visible; and 3) rES33-EITB to detect antibodies in serum [22], which was positive when the rES33 band was visible. For cysticercosis, two serum-based tests were used: 1) serum Ag ELISA to detect antigens [23] using a pre-determined protocol to determine the cut-off value, and 2) rT24H-EITB to detect antibodies [17], which was considered positive when the rT24H band was visible.”  “Further details regarding the reference tests are available in the study protocols [10,11].” |
|  |  | **12** | Definition of and rationale for test positivity cut-offs or result categories of **the tests under evaluation**, distinguishing pre-specified from exploratory | Section 2.3 and 2.4. “The TS POC is an antibody-detecting lateral flow assay, using two test strips, each with a previously characterised recombinant protein, rES33 for the TS POC T test strip and rT24H for the TS POC CC test strip [17,18]. During initial assessments conducted within laboratory settings using known positive and negative control sera, the TS POC test demonstrated encouraging results. The TS POC T test strip exhibited a sensitivity of 82% and a specificity of 99% in detecting taeniosis. In the case of NCC, the TS POC CC test strip had a sensitivity of 88%, and 93% for infection with multiple cysticerci, with a corresponding specificity of 99%. The TS POC cassette prototype was assembled at CDC, Atlanta.”  “For taeniosis, the following three tests were used: 1) copro Ag ELISA according to Allan et al. [23] and modified by Mwape et al. [24] to detect *Taenia* antigens in stool using a predefined cut-off value of 0.972; 2) copro mPCR according to Yamasaki et al. [7] to detect *Taenia* DNA in stool, which was considered positive only when a 720 bp *T. solium* band was visible; and 3) rES33-EITB to detect antibodies in serum [25], which was positive when the rES33 band was visible. For cysticercosis, two serum-based tests were used: 1) serum Ag ELISA to detect antigens with the cut-off value determined by comparing the optical density to that of a set of negative serum samples according to Dorny *et al*. [26], and 2) rT24H-EITB to detect antibodies [20], which was considered positive when the rT24H band was visible.”  “Further details regarding the reference tests are available in the study protocols [10,11].” |
|  |  | **13** | Whether clinical information was available to the performers or readers of **the tests under evaluation** | Section 2.4 Sample collection and testing: Samples arriving at the laboratories were only labelled with a pseudonymized code, so laboratory personnel performing the tests were blinded to the TS POC result and the participant’s cohort. |
|  | *Analysis* | **14a** | **BLCM model** for estimating measures of diagnostic accuracy | Pg 10, section 2.5.1. “Due to the lack of a gold standard test for infection with adult worm and larval stages of *T. solium*, diagnostic accuracy measures were estimated using a Bayesian Latent Class Model (BLCM)-like approach according to Berkvens et al. [24] using Open BUGS software version 3.2.3 (www.openbugs.net). The analysis allowed the sensitivities and specificities to differ conditional on the other test results. The analyses were performed for each of the three cohorts separately.” |
|  |  | **14b** | **Definition and rationale of prior information and sensitivity analysis** | Section 2.5.1.2.Priors: “For the probabilistic constraints in the Bayesian analyses, initially the same priors were used as described previously, for the evaluation of the TS POC test accuracy at community level (Mubanga, Trevisan, et al., 2021; Mubanga, Van Damme, et al., 2021). However, the priors were revised since diagnostic accuracy measures differ according to the target population, and the data did not support the priors in certain models, as demonstrated by high Bayesian P values (see S2 Appendix for all model outcomes). Therefore, new priors were defined based on the knowledge that was obtained about the test performance during the field studies at community level (Mubanga, Trevisan, et al., 2021; Mubanga, Van Damme, et al., 2021). Given the well-documented bias of sensitivity and specificity estimates from laboratory-based case-control studies, and the lack of field validation data, this approach was deemed the most appropriate. The outputs of the least restrictive models of the community-based studies were used as a basis to determine the new sets of priors and were updated after a more thorough literature search whenever necessary. The priors that were used during the first (initial priors) and second (new priors) round of analyses, including the rationale, are given in S2 Appendix.” |
|  |  | **15** | How indeterminate results **of the tests under evaluation** were handled | Section 2.5.1.1 Handling missing and inconclusive results: “The test strips of the TS POC test were read by two readers, and a third reader in case the first two readers disagreed. There were no inconclusive TS POC results found. For cysticercosis, participants with one or more missing blood results were excluded from the analysis, and for taeniosis, participants with a missing result for a blood test and/or stool test were excluded, i.e. a complete case analysis was performed.” |
|  |  | **16** | How missing data **of the tests under evaluation** were handled | Section 2.5.1.1 Handling missing and inconclusive results: “For cysticercosis, participants with one or more missing blood results were excluded from the analysis, and for taeniosis, participants with a missing result for a blood test and/or stool test were excluded, i.e. a complete case analysis was performed. To avoid partial verification bias due to the two-phase sampling, the multinomial probabilities in the models were adapted according to the observed sampling frequencies. The weighting was determined by the proportion of complete cases per test strip result.” |
|  |  | **17** | Any analyses of variability in diagnostic accuracy, distinguishing pre-specified from exploratory | Section 2.5.1.3. “Since rES33-EITB detects exposure (antibodies) whereas copro mPCR and copro Ag ELISA detect infection (parasitic DNA and proteins, respectively) and the TS POC T test was developed to identify active infections by detecting higher levels of antibodies, the analyses were also repeated without rES33-EITB to estimate the accuracy for detecting active infection. The estimates from models with and without rES33-EITB were very similar (see S2 Appendix), so only the models including rES33-EITB are reported as final model for taeniosis.” |
|  |  | **18** | Intended sample size and how it was determined | Section 2.5.3. “The sample sizes were calculated to obtain a desired precision of 10% around the sensitivity and specificity of the TS POC test, resulting in 600 individuals in cohort 1, and 2000 individuals, distributed over cohort 2 and 3 [11].” |
|  | **RESULTS** |  |  |  |
|  | *Participants* | **19** | Flow of participants, using a diagram | Figures 1, 2 and 3 |
|  |  | **20** | Baseline demographic and clinical characteristics of participants | Section 3.1 Study population. “In total, 3055 participants were recruited in three district hospitals in Tanzania. Participants were recruited for neurological signs/symptoms compatible with NCC (cohort 1, n = 742); complaints compatible with intestinal worm infection (cohort 2; n = 1661); and other symptoms (cohort 3; n = 663). The flows for each of the three cohorts are visualised in Figs 1-3, respectively. The TS POC test was performed in 601 participants of cohort 1, 1661 participants of cohort 2, and 662 participants of cohort 3. The baseline characteristics of these participants in each of the three cohorts are given in Table 1. The median age varied between 33 and 40 years and the proportion of women varied between 52% and 69%. The characteristics of patients who were selected for sampling, and of the participants in the final analysis set, are provided in S1 Appendix.” |
|  |  | **21** | **Not applicable: the distribution of the targeted conditions is unknown, hence the use of BLCM** |  |
|  |  | **22** | Time interval and any clinical interventions between **the tests under evaluation** | Section 3.3 Taeniosis tests: “Most participants provided a stool sample immediately after recruitment, with a median duration of two days between the TS POC test and processing of the stool sample in the hospital lab (based on 220 participants for whom both dates were known). Nevertheless, 27 participants provided a stool sample more than one month after recruitment.” |
|  | *Test results* | **23** | Cross tabulation of the **tests’ results (or for continuous tests results their distribution by infection stage)** | Tables 2 and 4 |
|  |  | **24** | Estimates of diagnostic accuracy **under alternative prior specification** and their precision (such as 95% **credible/probability intervals**) | Section 3.3.2. “The TS POC T test strip had an estimated sensitivity of 50.2% [95% CI 4.9 - 96.4] in cohort 1, 40.8% [2.2 - 95.2] in cohort 2, and 40.4% [2.3 - 95] in cohort 3. The specificity was 98.6% [97.1 - 99.6], 99.3% [98.7 - 99.7] and 99.4% [98.5 - 99.9], respectively.”  Section 3.4.2. “The sensitivity of the TS POC CC test strip was 77.5% [37.8 - 99.2] in cohort 1, 24.9% [6.4 - 52.7] in cohort 2, and 44.2% [2.3 - 95] in cohort 3. The specificity was 92.3% [86.5 - 98.8], 99.1% [97.8 - 100], and 98.1% [96.1 - 99.7], respectively.”  The outcomes of all the models that were used is provided in S2 Appendix. |
|  |  | **25** | Any adverse events from performing **the tests under evaluation** | Section 2.4 “No adverse events were recorded during the collection of blood samples for the serological tests.” |
|  | **DISCUSSION** |  |  |  |
|  |  | **26** | Study limitations, including sources of potential bias, statistical uncertainty, and generalisability | Discussion. “We used several strategies to minimize bias in this diagnostic accuracy study [13,14]. Despite these efforts, certain factors may have affected the sensitivity and specificity estimates. One potential source of bias for the taeniosis evaluation is the time interval between the initial test and the stool sample collection, which could result in disease progression bias. Although most participants submitted a stool sample within two days of the TS POC test, some waited considerably longer to return a stool sample, raising the possibility of infection acquisition or changes in egg excretion during the interval. In contrast, blood samples were collected promptly by a nurse, thus limiting the risk of disease progression bias. Logistical issues, such as sample shipment and COVID-19-related delays, may have further affected test outcomes. Prolonged storage could have led to false negatives and variations in the stability of antibodies, antigens, and DNA, could have contributed to the observed low agreement between tests. Our two-stage design, sampling all POC test positives and a subset of negatives, aimed to reduce the number of tests. Although weighted adjustments were applied to address partial verification bias, the subset of verified negatives (every 10^th^ patient), may not be fully representative because of the systematic sampling and missing stool or blood samples. Recently, a point-of-care test for the detection of antigens in urine has undergone laboratory validation, showing promise for future field validation [31]. Urine is less invasive than blood sampling and more practical to collect than stool samples, offering potential advantages. The TS POC CC test result was included in the model for taeniosis to account for the two-stage design, but may have influenced results, and complicates the interpretation of the latent class. Due to the lower sensitivity of the TS POC test than the preliminary laboratory tests, we only identified few infections, particularly for taeniosis, which resulted in wide credible intervals for sensitivity. The results should be interpreted with caution as the smaller-than-anticipated analysis population in this study may have affected the reliability and precision of the estimates obtained. Moreover, transforming continuous variables, such as antibody levels or DNA concentrations, into binary outcomes (presence or absence of infection) inevitably leads to a loss of valuable information. This simplification can result in misclassification, particularly near cut-off thresholds.”  “In our study, a substantial number of patients were excluded from cohort 1 due to incorrect application of the criteria for severe progressive headache during recruitment. Consequently, the diagnostic estimates may not accurately reflect patients with a presentation of severe progressive headache that differ from the criteria used during screening, potentially limiting the generalizability of our findings to broader patient populations.”  “The lack of data on test performance for cysticercosis detection made selecting prior information challenging. Priors were updated based on community-level evaluation data [16], and were chosen to be minimally restrictive, contributing to wide credible intervals.” |
|  |  | **27** | Implications for practice, including the intended use and clinical role of **the tests under evaluation in relevant settings (clinical, research, surveillance etc.)** | Discussion. “The primary objective of this study was to assess the sensitivity and specificity of the TS POC test under field conditions in people attending district hospitals in Tanzania for the detection of taeniosis and cysticercosis. The study lateral-flow test demonstrated a moderate sensitivity for cysticercosis detection in individuals suspect of (neuro)cysticercosis. In contrast, the sensitivity was largely unsatisfactory for the detection of taeniosis even in clinical suspects. The relatively high specificity of the test may be useful in clinical care.”  “… Despite these limitations, this study showed valuable insights in diagnostic tests for *T. solium* infections in hospital settings. Its main strength was evaluating the test in target populations, yielding more realistic estimates than diagnostic case-control studies conducted in laboratory settings.”  “Rapid diagnostic tests can be particularly useful for identifying *T. solium* taeniosis, as they shorten diagnostic turnaround times and enable quicker treatment, preventing the spread of infectious eggs. A high sensitivity is essential to avoid missing infected individuals. In our study, the TS POC T test strip had a sensitivity of 40% to 50%, with wide credible intervals, falling short of the 95% sensitivity threshold recommended in Target Product Profiles (TPPs) for monitoring of control interventions, as well as for diagnosis and treatment [8]. Additionally, rES33-EITB, copro Ag ELISA and copro mPCR also showed sensitivities below the 95% TPP threshold, highlighting the need for improved diagnostic methods validated under field conditions.”  “Despite the potential presence of comorbid infections in our study populations, the specificity of the TS POC test seems rather high, and may be useful for epidemiological studies and monitoring of interventions. Nevertheless, when the disease prevalence decreases, a very high specificity is required [41], so the specificity of the tests would have to be evaluated in post-intervention populations to determine if it is fit for purpose.” |
|  | **OTHER INFORMATION** |  |  |  |
|  |  | **28** | Registration number and name of registry | “The trial was registered at the Pan African Clinical Trials Registry with identifier PACTR201712002788898.” |
|  |  | **29** | Where the full study protocol can be accessed | “A comprehensive description of the trial rationale, design and methodology can be found in Trevisan et al. [11].” |
|  |  | **30** | Sources of funding and other support; role of funders | “Funding for this research was provided by the European & Developing Countries Clinical Trials Partnership (grant number DRIA2014-308) and the German Federal Ministry of Education and Research (grant number 01KA1617) as part of the research grant titled "Evaluation of an antibody detecting point-of-care test for the diagnosis of *Taenia solium* taeniosis and (neuro)cysticercosis in communities and primary care settings of highly endemic, resource-poor areas in Tanzania and Zambia, including training of and technology transfer to the Regional Reference Laboratory and health centers (SOLID)". The funders of this research had no role in this study.”  Acknowledgements “We extend our gratitude to all the dedicated hospital staff members and the willing participants for their invaluable contributions to this study. Special recognition is also due to the laboratory technicians, Sandra Vangeenberghe, Maxwell Masuku, Chembe Mwelwansofu, Anke Van Hul, Ana Lucia Fajardo for their diligent analysis of the samples.  Special thanks are due to the members of the SOLID External Advisory Board for their guidance, and Helena Ngowi and Maria V. Johansen for their valuable contributions during the initial phase of this study.” |
|  |  |  |  |  |

STARD - BLCM

STARD-BLCM stands for “Standards for the Reporting of Diagnostic accuracy studies that use Bayesian Latent Class Models” and is a modification of the STARD statement (which was recently updated to STARD2015). STARD-BLCM aims to facilitate improved quality of reporting for diagnostic accuracy studies that use Bayesian latent class models in the absence of a reference standard. The proposed modifications are relevant to both Bayesian and frequentist estimation methods but the focus is on the former.

More information for STARD (STARD2015) can be found at: [http://www.equator-network.org/reporting-guidelines/stard](http://www.equator-network.org/reporting-guidelines/stard/)

More information for STARD-BLCM can be found at: [http://www.equator-network.org/reporting-guidelines/stard-blcm](http://www.equator-network.org/reporting-guidelines/stard-blcm/)
