# Supplementary material for: Evaluation of a rapid lateral flow assay for the detection of taeniosis and cysticercosis at district hospital level in Tanzania: A prospective multicentre diagnostic accuracy study
Source: PLoS Negl Trop Dis. 2025 Mar 28;19(3):e0012310. doi: 10.1371/journal.pntd.0012310 (PMC11977959; doi:10.1371/journal.pntd.0012310)
Supplement: Attachment — Submitted filename: Response to Reviewers.docx [file pntd.0012310.s006.docx]

# Response to Reviewers

## Methods

Reviewer #1:The study’s objectives—assessing the diagnostic performance of the TS POC test—were clearly articulated, with a testable hypothesis explicitly stated, ensuring a focused and compelling study. Given the absence of a reference test for detecting T. solium taeniosis and cysticercosis, the use of the Bayesian Latent Class Model is a valid and appropriate choice for estimating diagnostic accuracy. The sample size calculation, as detailed in previous publications, was properly estimated. However, both the prevalence of T. solium and the sensitivity of the test’s performance under laboratory conditions were overestimated, leading to a relatively small number of positive cases for analysis. This issue was appropriately acknowledged by the authors in the discussion section. Regarding the statistical methodology, I recommend that the authors further clarify and define the targeted the targeted condition (latent class) in the models used for the two tests. For example, it is unclear why the TS POC CC test for cysticercosis is included in the model designed to estimate taeniosis. This inclusion affects the latent class and, consequently, the sensitivity results of the TS POC test under evaluation, which is intended to diagnose taeniosis alone. Similarly, the exclusion of the rES33-EITB test, which targets antibodies, is not sufficiently justified, particularly since the TS POC T test—also targeting antibodies—was included in the model. The use of directed acyclic graphs, as recommended in the recent paper by Denwood (doi: 10.1371/journal.pntd.0012481), could enhance clarity and assist readers in understanding the model structure.

Reply:

Thank you for the thorough review and appreciation. The TS POC CC test was included in the models to account for the two-stage design that was used in the study. The selection of participants was based on the positivity of TS POC T and TS POC CC test strip (all positives were selected for sampling, and 10% of negatives). In order to be able to do a correct weighing (since there was a relatively large proportion of TS POC CC positive participants within the selected subgroup, this is important to do), the TS POC CC test results had to be included in the model. Moreover, while the TS POC CC test is primarily intended for cysticercosis detection, the results of the test strip seem to be related seems to be related to TS POC T positivity , which justified its inclusion in the model.

This is in the Material and Methods section: ”For the evaluation of the TS POC T strip, the TS POC CC test strip result was included in all models to account for the relatively large number of TS POC CC positive participants within TS POC T negative complete cases.”

The TS POC CC test is now also included in the DAG. We acknowledge, however, that including the TS POC CC test in the model could influence the estimates. We therefore included this limitation in the discussion as follows:

”The TS POC CC test result was included in the model for taeniosis to account for the two-stage design, but may have influenced results, and complicates the interpretation of the latent class.”

The exclusion of the rES33-EITB to estimate the diagnostic accuracy of taeniosis (presence of the tapeworm, i.e. active infection, by using only stool-based tests) is justified because the TS POC T test was primarily developed to detect active infection (and not only exposure/past infection) by detecting higher levels of antibodies (because for previous infections/exposure, lower levels are expected). Therefore, we think for the model without rES33-EITB, infection (presence of the tapeworm in the intestines) is the latent class. We clarified this in the paper in section 2.5.1.3 Models:

”Since rES33-EITB detects exposure (antibodies) whereas copro mPCR and copro Ag ELISA detect infection (parasitic DNA and proteins, respectively) and the TS POC T test was developed to identify active infections by detecting higher levels of antibodies, the analyses were also repeated without rES33-EITB to estimate the accuracy for detecting active infection.”

We appreciate the suggestion to incorporate DAGs to improve clarity. As suggested, two directed acyclic graphs (one for taeniosis and one for cysticercosis) were included in the supplementary materials to illustrate the latent class and clarify the relationships between the tests. This will help readers better understand the assumptions underlying the model and the interdependencies between the tests.

The DAG for taeniosis is included in section 1.3 of S2 Appendix:

The DAG for cysticercosis is included in section 2.3 of S2 Appendix:

Reviewer #2:
- Are the objectives of the study clearly articulated with a clear testable hypothesis stated? YES
- Is the study design appropriate to address the stated objectives? YES
- Is the population clearly described and appropriate for the hypothesis being tested? YES
- Is the sample size sufficient to ensure adequate power to address the hypothesis being tested? NO. In this study, the analysis populations appear to be very small (much smaller than the sample size estimate) making results very uncertain. When disease prevalence is very low and the number of disease positives is small, as in the present study, the data may not be strong enough to "override" the priors, so the choice of priors can have a significant impact on the results. In low prevalence situations and with 3-4 tests, sample sizes well above 1000 are recommended for reliable estimation. I recommend revisiting if the data allows BLCM.

Reply: we agree with this limitation, and we believe that we’ve addressed this limitation in an honest way on several occasions in our manuscript. We now also stated this more thouroughly in the discussion:

”Due to the lower sensitivity of the TS POC test than the preliminary laboratory tests, we only identified few infections, particularly for taeniosis, which resulted in wide credible intervals for sensitivity. The results should be interpreted with caution as the smaller-than-anticipated analysis population in this study may have affected the reliability and precision of the estimates obtained. Moreover, transforming continuous variables, such as antibody levels or DNA concentrations, into binary outcomes (presence or absence of infection) inevitably leads to a loss of valuable information. This simplification can result in misclassification, particularly near cut-off thresholds. Despite these limitations,...”

- Were correct statistical analysis used to support conclusions? In theory yes but given the actual data rather not for reasons as in previous point.
- Are there concerns about ethical or regulatory requirements being met? NO

2 Material and methods 2.1 Study design l. 146: You introduce the other tests as reference tests though in absence of a gold standard, which would in fact be the reference test, I suggest changing the terminology to avoid confusion. In line with the need for using the BLCM due to the absence of a gold standard, i.e., reference test, none of the other tests can be called reference, and they are equally handled in the model as the TS POC.

Reply: we agree, this was changed throughout the manuscript.

l. 148: Same here. I suggest cross-checking with the recommended terminology in the literature. The way it is phrased here it sounds as if the remaining tests form a composite reference standard but this would be in contrast to why you chose a BLCM.

Reply: we agree, this was changed throughout the manuscript.

2.4 Sample collection and reference testing Same comment regarding the use of the terminology of reference test which is usually reserved for the gold standard or composite reference standard.

Reply: we agree, this was changed throughout the manuscript.

l.216-218: I suggest moving the details on the test that was not used for this paper to discussion section instead of methods.

Reply: we prefer to keep it in the methods section as the same problem occurred in the community-based study in Zambia (Mubanga et al.).

2.5.1.1 Handling missing and inconclusive results l. 247-250: The method for addressing the potential partial verification bias analytically sounds very good. You may consider adding to your limitations that even weighted adjustments might not completely remove bias since the subset selected for further testing is not truly random (due to selecting every 10th patient) though this is only a very small risk in your case. Still, the effectiveness of the correction depends on how representative the subset of verified negatives is.

Reply: We acknowledge that the subset of verified negatives is not entirely random due to the systematic selection of every 10th patient. We have added this consideration to the limitations in the discussion section to clarify the potential impact:
"Although weighted adjustments were applied to address partial verification bias, the subset of verified negatives (every 10^th^ patient), may not be fully representative because of the systematic sampling and missing stool or blood samples."

2.5.3 Sample size and descriptive statistics The sample sizes are tailored to the diagnostic accuracy estimations though the actual analysis populations for BLCM are far below these numbers. Datasets may be to small to apply BLCM that produce reliable estimates and I recommend critically reflecting on it and at the minimum add a paragraph on the implications for interpretation and limitations in your discussion section.

Reply: We acknowledge that the actual analysis populations for the Bayesian analysis are smaller than the initially intended sample sizes for diagnostic accuracy estimations. We agree that smaller datasets may limit the reliability of the resulting estimates.

We already included this limitation on several occasions in the discussion:

- In the second paragraph of the discussion: ”However, due to the lower sensitivity of the TS POC test than the preliminary laboratory tests, we only identified few infections, particularly for taeniosis, which resulted in wide credible intervals for sensitivity”.
- In the third paragraph of the discussion: ”In our study, the TS POC T test strip had a sensitivity of 40% to 50%, with wide credible intervals, falling short of...” ... ”The low disease prevalence complicates sensitivity assessment, as seen in our cohorts where true prevalence was very low and similar to 0.6% [28] and 1.8% [12] in rural communities of Eastern Province of Zambia. Achieving a sensitivity above the 95% TPP threshold for taeniosis tests under field conditions with current tests and low prevalence would thus require very large studies to achieve accurate field validation.

To address this point even more, we have added the following to the discussion section, further highlighting this limitation:

"...The results should be interpreted with caution as the smaller-than-anticipated analysis population in this study may have affected the reliability and precision of the estimates obtained. Moreover, transforming continuous variables, such as antibody levels or DNA concentrations, into binary outcomes (presence or absence of infection) inevitably leads to a loss of valuable information. This simplification can result in misclassification, particularly near cut-off thresholds.”

Reviewer #3:

1. Please can the authors elaborate on the third cohort for testing indicated in lines 157 – 159? It is not apparent my this would be a target population for using the TS POC test.

Reply: The third cohort, consisting of individuals presenting with symptoms other than epilepsy/severe progressive headache or intestinal worm infections, was included because taeniosis patients do not always exhibit specific symptoms. This cohort represents a more general population of people attending district hospitals. The TS POC test was evaluated for its potential use in epidemiological studies among individuals attending district hospitals.

2. Are the criteria stated in lines 167 – 168 available in the appendix?: “Different additional criteria were used to be included in the different cohorts.” And this sentence should be rephrased (with reference to the additional criteria) to something similar to the below to improve clarity: “Different additional exclusion criteria were used in the different cohorts.”

The criteria were already described in the subsequent paragraph, but we agree that this could be clarified to improve readability. We have revised the sentence as follows to explicitly link the additional inclusion criteria to the description provided:

”Different additional inclusion criteria were applied to define eligibility for the different cohorts, as described below.”

3. Can the authors state what cut-off was used in line 210: “using a predefined cut-off value”.

Reply:”using a predefined cut-off value” was to clearly define the cut-off as pre-specified (vs exploratory) as requested in the STARD guidelines. We now included the value that was used (0.972) in the text.

4. I am not sure what you mean by “using a pre-determined protocol to determine the cut-off value” on line 214-215.

Reply: Similar as above, this was included to clarify that the cut-off value was determined according to a protocol. In this case, the cut-off value was determined by comparing the optical density (OD) of each serum sample with that of a set of negative pig serum samples (n = 8). A threshold was established at a probability due to chance (P) < 0.001, following the method described by Sokal and Rohlf (1981). This procedure is explained in reference 23.

Sokal, R. R., Rohlf, F. J., 1981. Biometry, 2nd Edition. Freedman and Company, New York.

The text in the manuscript was revised: ”...serum Ag ELISA to detect antigens with the cut-off value determined by comparing the optical density to that of a set of negative serum samples according to Dorny *et al*. [23]”

5. The authors state that on lines 216-218: “Although also the LLGP-EITB was initially planned to be included in the study [11], the results of this in-house test could not be used due to doubts about the validity of the test results [13]”. Reviewing reference [13], Mubanga et al. elaborate that “LLGP EITB….they did not satisfy the internal quality control during the laboratory analyses”; can the authors confirm that this was the same issue? The LLGP-EITB is a major diagnostic used for human CC, especially in central/south American settings, so to not include this diagnostic as part of the reference testing needs further justification.

Reply: We confirm that the LLGP-EITB was not included in our study due to the problems encountered with its implementation in Zambia, as described by Mubanga et al. [13]. The LLGP-EITB did not satisfy internal quality control standards during laboratory analyses, so it was not feasible to use the results of this diagnostic test in our study.

We clarified this in the text, which now reads as follows:

”Although also the LLGP-EITB was initially planned to be included in the study [11], this in-house test could not be used due to doubts about the validity of the test results. The test did not satisfy internal quality control standards during laboratory analyses [13], so it was decided to exclude the results to ensure the reliability of the results presented in this study.”

6. Where attempts made to access any neuroimaging based results, since the patient population was derived from a district hospital setting, for the validation of the TS POC CC output (against active infection, rather than just exposure?).

Reply: Yes, neuroimaging was performed for a selection of these patients to validate the TS POC test for neurocysticercosis (NCC). Also a subgroup analysis was performed to assess the performance of the test to detect patients with active cysticercosis infections. These findings have been published in detail by Stelzle et al., <https://doi.org/10.1016/S1473-3099(23)00378-X>. Our current paper only focusses on taeniosis and cysticercosis (not limited to cysticerci in the brain, but anywhere in the body). The background explaining the focus of this paper within the project is explained in the introduction:

“…To evaluate its real-world performance in resource-poor, highly endemic areas in sub-Saharan Africa, studies were designed to evaluate the performance characteristics of the TS POC test in two different settings: in rural communities in Zambia [13], and in district hospitals in Tanzania [14]. The evaluation of the TS POC test for the detection of *T. solium* taeniosis and cysticercosis at community level has been reported elsewhere [15,16]. Also the evaluation of the TS POC test for the neuroimaging-based diagnosis of NCC in both settings were reported elsewhere [17,18], and are thus out of the scope of this paper. The focus of this paper is the evaluation of the TS POC test for the detection of infection with the adult tapeworm (taeniosis) and/or the larval stage (cysticercosis) of *T. solium* in individuals attending district hospitals (Tier 2 level) in Tanzania.”

7. Can the authors explain why this statement is necessary on line 219: “No adverse events were recorded”, is this in relation to the test itself or taking blood for the test?

Reply: This statement is included as a requirement of the STARD-BLCM reporting guidelines, specifically item 25: "Report any adverse events from performing the tests under evaluation." In this study, the adverse event reporting relates to the process of taking a blood sample for the serological tests. We have clarified this in the revised manuscript.

We have updated the sentence to: "No adverse events were recorded during the collection of blood samples for the serological tests."

8. Regarding the statement on lines 254 – 256: “However, the priors were revised since diagnostic accuracy measures differ according to the target population, and the data did not support the priors in certain models”; With the priors being revised based on the least restrictive set of posteriors generated from the community-based study, it is not clear how these would be relevant for the hospital-based setting (even using the least-restrictive set of posteriors). Can the authors expand on why they think this approach is suitable.

Reply: The only prior knowledge available for the TS POC test was derived from the results obtained during the community-based study conducted in Zambia. While we acknowledge the differences between community- and hospital-based settings, the absence of alternative robust field validation data necessitated this approach. Estimates for sensitivity and specificity derived from laboratory-based case-control studies are well-known to be biased and not reflective of real-world performance. Additionally, for cysticercosis (CC), most available data pertain to neurocysticercosis (NCC), which is a different disease manifestation and thus not suitable as prior information for this test.

To clarify this, we added to section 2.5.1.2 Priors: ”Given the well-documented bias of sensitivity and specificity estimates from laboratory-based case-control studies, and the lack of field validation data, this approach was deemed the most appropriate.”

9. It would still be useful to include the model outputs from the combined cohorts (indicated on lines 274 – 276) in the supplementary, even with high Bayesian p values for completeness.

Reply: While we understand the value of providing model outputs for completeness, including the outputs from the combined cohorts in the main manuscript is not appropriate due to the poor model fit. The high Bayesian p-values indicate a lack of fit, which makes the results unreliable and potentially misleading. We therefore include a reference to the appendix, where the outputs of these models are shown, along with an explanation of the poor fit. This ensures transparency while avoiding the dissemination of results that do not meet the required standards of reliability.

10. Please can the authors explain why a hierarchal model was not used, to account for hierarchical structure of the data (especially that patients were recruited from three different district hospitals, where systematic differences e.g. healthcare infrastructure, patient population, or diagnostic practices may exist?)

Reply: While we recognize the potential advantages of using a hierarchical model to account for the structure of the data, this approach was not feasible due to the limited data available.

## Results

Reviewer #1:The results are well presented and, with the supplementary materials provided in the appendix, are comprehensive and thorough.

Reply: Thank you for your kind feedback. We are pleased that you found the results well-presented and the supplementary materials comprehensive and thorough.

Reviewer #2:

I recommend to use the actual analysis populations as basis for the descriptive Table 1 or at least first describe the full cohorts and in the same table also describe the characteristics of the complete case analysis populations. This way you can assess and discuss selection bias that may have been introduced by the strong reduction of the initial cohorts to those individuals that had complete diagnostic test results across all tests under evaluation. I thus also recommend moving Table 1 below the Fig. 1-3 so people already understand the flow of participants.

Reply: Thank you for this suggestion. We understand the importance of assessing and discussing potential selection bias introduced by the reduction of the initial cohorts to individuals with complete diagnostic test results. However, incorporating the characteristics of the complete case analysis populations alongside the full cohorts in Table 1 would not be correct to interpret potential selection bias due to the two-stage design of the study. In complete cases, a relatively higher proportion of TS POC-positive participants are included. Serological results (and thus selection of patients) can depend on factors such as gender and age, so population characteristics can differ when comparing the initial population to the complete cases, even without selection bias. Moreover, the complete cases for taeniosis evaluation are different from those for cysticercosis evaluation, further complicating the table and interpretation. Correct interpretation of the results would require some knowledge about the design to avoid wrong conclusions, and it requires a much larger table while adding only limited extra information. We decided not to include the table in the main manuscript, but included two tables in S1 Appendix (S1 Table 1C for taeniosis and S1 Table 1D for cysticercosis) with the summary statistics for non-selected patients, selected/non-complete cases, and complete cases.

Table 3: It is recommended to add 95% CI to the estimates.

Reply: We agree that including 95% CI is valuable for understanding the uncertainty of estimates. However, due to the need to apply weighting to account for the two-stage design, calculating the 95% CIs is not straightforward. We acknowledge the importance of addressing uncertainty, particularly given the low number of test positive cases in our study. This concern is already reflected in the manuscript, where we clearly highlighted this: “The point estimates for agreement and positive agreement were low among all tests for taeniosis (Table 3). Due to the low number of test positive samples, the estimates for agreement should be interpreted with caution.”

General comments to BLCM models: You may reconsider using BLCM for your data in general. The underlying analysis populations and test positivity rates are very low causing a large uncertainty regarding all estimates. This is an indication that the model estimates are instable and e.g., senstitivities almost across the whole range of values from 0-100 are also plausible results given your data. Please critically revisit if the data is suitable to run BLCM. The choices of priors are very restrictive and seem to dominate the model outputs regarding specificities almost fully ignoring the underlying data. Were the tests all considered to be conditionally independent or dependent? Please specify your assumptions on the dependence structure in the methods section and clarify how it was handled in the analysis.

Reply: We acknowledge that the small size of the analysis populations and the low test positivity rates contribute to a high degree of uncertainty in the estimates. This limitation was recognized during the analysis and is explicitly discussed in the manuscript. Despite this, we believe that BLCM remains the best available method for our study (with sensitivity and specificity of the TS POC test as primary endpoints), given the lack of a gold standard test.

We recognize the influence of the priors on the model outputs. The choice of priors was informed by existing evidence and designed to reflect the available knowledge. For sensitivity, we used minimally restrictive priors. Specificity priors were more constrained, though we don’t think they had a large influence on the results of our primary endpoints (sensitivity and specificity of the TS POC test strips) since we had many test negative results. For example, for the specificity of the TS POC T test, a prior of 95-100% was used, but we don’t think this has a large effect on the posterior. Since (for example) in cohort 2, only 10 out of 1661 participants tested TS POC positive, even if these 10 participants would be all false positive (worst case), the specificity would be (at least) 99.4% in this cohort. Also for the TS POC CC specificity, which was estimated to be the lowest in cohort 1, the same conclusion can be made: 102 out of 601 tested individuals tested TS POC CC positive in cohort 1, so worst case (if we assume all of these were false positive, which is not the case), the specificity would be 83%, showing that the lower level of the prior for specificity (80%) in this cohort was very reasonable.

The analysis allowed the sensitivities and specificities to differ conditional on the other test results, as mentioned in section 2.5.1.

Reviewer #3:

In terms of those individuals not included from the pool of “Potentially eligible patients” in cohort 1, due to “incorrect headache criteria used”, the constitutes a reasonably large proportion (138/742) – can the authors expand on this limitation, especially in the discussion.

Reply: Thank you for pointing this out. We agree that the exclusion of a substantial proportion of potentially eligible patients due to the use of incorrect headache criteria represents a noteworthy limitation. To address this, we have expanded on this issue in the discussion section, considering its potential implications for the study findings.

In the discussion section, we have added the following sentences:
"In our study, a substantial number of patients were excluded from cohort 1 due to incorrect application of the criteria for severe progressive headache during recruitment. Consequently, the diagnostic estimates may not accurately reflect patients with a presentation of severe progressive headache that differ from the criteria used during screening, potentially limiting the generalizability of our findings to broader patient populations.”

It would be useful in Tables 2 and 4 to include the number of results under the first 4 columns, especially where there are negative/positive combinations.

Reply: It’s not clear which numbers should/could be added under these columns since the first 4 columns represent the test result categories (positive or negative), hence each row of the table represents the different test result combinations. The numbers in the next columns represent the number of participants for each test result combination. The total number of participates per result combination is also included, in the column ’Total’. In case we misunderstood, please clarify which numbers should be added.

## Conclusions

Reviewer #1:The authors clearly acknowledge the study's methodological limitations in the discussion section and carefully consider them when formulating their conclusions.

Reply: Thank you for your positive feedback.

Reviewer #2:

- Are the conclusions supported by the data presented? BLCM estimates are very uncertain which makes evidence on diagnostic accuracy of the tests based on the empirical data rather weak.

- Are the limitations of analysis clearly described? NO, limitations of the methods applied and the scarce data and low prevalence are not sufficiently adressed.

- Do the authors discuss how these data can be helpful to advance our understanding of the topic under study? YES

- Is public health relevance addressed? YES

Reply: We acknowledge the limitations of the data and the model, and hope that our replies and revisions make the manuscript appropriately cautious and transparent about the constraints of the study.

Reviewer #3:This section is well addressed - but see specific comments relating to the Discussion and Conclusion sections under "Summary and General Comments"

Reply: Thank you for your encouraging feedback regarding the conclusions section. We have reviewed and addressed your specific comments above.

## Editorial and Data Presentation Modifications

Reviewer #1:Minor Revision I suggest that the authors further clarify the target condition underlying the models to improve the interpretation of the diagnostic performance estimates.

Reply: The DAGs were included in appendix to make it clearer.

Reviewer #2:(No Response)

Reviewer #3:The data and outputs are generally well presented, I include some minor recommendations in the "Results" section.

## Summary and General Comments

Reviewer #1:(No Response)

Reviewer #2:

Methods The number of diagnostic tests should be part of the abstract already. How many coprological and serological tests were performed in parallel per patient? This is informative to later interpret results from the BLCM. Also already mention that you assessed your BLCM varying the prior specifications.

Results Are the populations in whom the TS POC was conducted also complete for the other tests? Please specify N of analysis populations for BLCM as that appears to be the core of the manuscript. The section only focusses on TS POC results though other tests performance was assessed too. Will be good to mention the others performance too. Add info on what is represented in square brackets, 95% Credible Intervals?

Reply: Thank you for your suggestions. While we agree that including the number of diagnostic tests performed per patient and the variations in prior specifications would provide valuable context, it is not feasible to include all this information within the word limit of the abstract.

In this clinical trial, the primary endpoints were the sensitivity and specificity of the newly developed TS POC test. Although the Bayesian analysis provides additional outputs, these were considered exploratory endpoints and are addressed in detail within the Results section of the manuscript. The study has three different cohorts of patients, and two different disease presentations (taeniosis and cysticercosis), so including sensitivity and specificity results of all different tests of the 6 different models is unfortunately not possible. Due to the word limit, we have to restrict the information in the abstract while still including the necessary elements for reporting to adhere to the guidelines, STARD for abstracts.

Discussion I suggest replacing ‘suboptimal sensitivity’ by ‘low sensitivity’ since 5 out of 6 estimates are <50%. I suggest rephrasing the last sentence where you recommend ‘for the development and validation of better diagnostic tests’ by sth like ‘to enhance and validate these tests for better performance in practical, real-world settings’.

Reply: These were revised as suggested.

Background l. 123-129: I suggest moving most of what you have not done to the discussion and rather focus on the objectives of this paper as described from l. 130 in the background section. Please mention which other tests are being assessed along with the TS POC which is your main interest.

Reply: Thank you for the suggestion. However, since the data presented in this manuscript are part of a larger project (in different countries/settings and for the evaluation of the test for different disease presentations), we think this context is necessary to understand the focus of this paper.

Reviewer #3:

This is an important study, with a suitable methodology to address the problem. I have outlined a few points of clarification required for the methods before I would suggest this manuscript is ready for acceptance/minor revisions. Other comments include for specific sections (generally minor comments):

Introduction

Major 1. It is not clear to me how this study differs from reference 11 which is also a study of the diagnostic performance in district hospitals (level 2?) in Tanzania as reported in the statement (lines 125 – 126): “To evaluate its real-world performance in resource-poor, highly endemic areas in sub-Saharan Africa, the performance characteristics of the TS POC test were evaluated in two different settings: …….., and in district hospitals in Tanzania [11]” Please can the authors make this clarify and make the distinction clearer in the introduction.

Reply: reference 11 is a reference to the study protocol of the study in hospital settings in Tanzania. Reference 11 doesn’t contain any results. The introduction was revised to make this more clear:

”To evaluate its real-world performance in resource-poor, highly endemic areas in sub-Saharan Africa, studies were designed to evaluate the performance characteristics of the TS POC test in two different settings: in rural communities in Zambia [10], and in district hospitals in Tanzania [11]. The evaluation of the TS POC test for the detection of *T. solium* taeniosis and cysticercosis at community level has been reported elsewhere [12,13]. Also the evaluation of the TS POC test for the neuroimaging-based diagnosis of NCC in both settings were reported elsewhere [14,15], and are thus out of the scope of this paper. The focus of this paper is the evaluation of the TS POC test for the detection of infection with the adult tapeworm (taeniosis) and/or the larval stage (cysticercosis) of *T. solium* in individuals attending district hospitals (Tier 2 level) in Tanzania.”

Minor

1. On lines 100-101, it would be useful to include the prevalence ranges from refs 1 and 2: “In Tanzania, the prevalence of T. solium taeniosis and cysticercosis is high [1,2]”

Reply: we revised the sentence as follows:

"In Tanzania, the prevalence of T. solium taeniosis and cysticercosis is high, with prevalence rates of up to 5% in stool and 45% having antibodies based on serology, respectively [1,2], resulting in significant public health and economic consequences [3]."

2. Please provide references for examples of different infectious diseases to support this statement on lines 112 – 113: “Rapid diagnostic tests (RDTs) have revolutionized the field of diagnostics by providing fast and easy-to-use tools for the detection of diseases in resource-limited settings”

Reply: We’ve added the following sentence to the introduction: ”The implementation of RDTs has the potential to significantly reduce the global burden of disease, as evidenced by studies estimating the impact on acute lower respiratory infections [9], human immunodeficiency virus[10], and tuberculosis [11].”

3. Please provide a reference to support the following statement on lines 121-123: “The test showed a promising sensitivity and specificity to detect T. solium taeniosis and NCC during its preliminary evaluation under laboratory conditions”

Reply: The initial evaluation of the diagnostic accuracy of the TS POC test under laboratory conditions hasn’t been published.

Methods

Minor 11. A brief sentence or two to explain the overall trial how the diagnostic study relates to the overall trial, after lines 137 – 138, would be useful.

Reply: The relation of this study in the overall trial was described above, in the introduction: ”To evaluate its real-world performance in resource-poor, highly endemic areas in sub-Saharan Africa, studies were designed to evaluate the performance characteristics of the TS POC test were evaluated in two different settings: in rural communities in Zambia [13], and in district hospitals in Tanzania [14]. The evaluation of the TS POC test for the detection of T. solium taeniosis and cysticercosis at community level has been reported elsewhere [15,16]. Also the evaluation of the TS POC test for the neuroimaging-based diagnosis of NCC in both settings were reported elsewhere [17,18], and are thus out of the scope of this paper. The focus of this paper is the evaluation of the TS POC test for the detection of infection with the adult tapeworm (taeniosis) and/or the larval stage (cysticercosis) of T. solium in individuals attending district hospitals (Tier 2 level) in Tanzania.”

12. Please replace “neither” with “either” in line 147 to remove the double-negative in the sentence.

Reply: This was corrected as suggested.

13. Please provide a reference for the statement on lines 189 – 194 or if references 17 and 18 are relevant, please use the references here in addition: “During initial assessments conducted within laboratory settings using known positive and negative control sera, the TS POC test demonstrated encouraging results. The TS POC T test strip exhibited a sensitivity of 82% and a specificity of 99% in detecting taeniosis. In the case of NCC, the TS POC CC test strip had a sensitivity of 88%, and 93% for infection with multiple cysticerci, with a corresponding specificity of 99%.”

Reply: The results of the initial laboratory evaluation of the TS POC test haven’t been published.

14. Please change “nor” to “or” on line 207 to avoid the double negative.

Reply: This was changed as suggested.

15. Please change “is” to “are” on line 265

Reply: This was changed as suggested.

Results

Minor 1. In reference to 3.3.2 and 3.4.2 in estimation of diagnostic performance, these sections are based on the BLCM modelling? Please can the authors state this to link the results more clearly to the methods section.

Reply: This was added to both sections.

Discussion

Major 1. Can the authors explain why they think the sensitivity of the Copro-Ag ELISA is higher across the cohorts compared to the Copro mPCR molecular test?

Reply: Given the wide and overlapping credible intervals, and considering that the sensitivity and specificity of the other tests are exploratory outcomes of this trial, we decided not to explore this in detail. However, it is worth noting that only three complete cases tested positive using the copro mPCR method, compared to twelve patients using the Copro-Ag ELISA in our study.

2. The authors could expand on the usefulness of the tests in ruling out disease (with a negative test) given the high specificity and the applicability related to this.

Reply: We agree that the high specificity could make the tests useful for ruling out disease; however, the low sensitivity limits their applicability in this context. Therefore, we opted not to expand further on this aspect in the discussion.

3. While the samples were shipped and processed in Belgium, which the authors suggest could raise the issue of increased false negatives, can the authors indicate why it was not possible to process samples in-country, presumably due to limited infrastructure, and therefore what attempts are being made to strengthen capacity in-country to support processing of samples that require lab processing?

Reply: At the time of the study, it was not possible to process the samples in-country due to limited laboratory capacity. The analyses were conducted more than four years ago. Since then, infrastructure in the country has improved, and samples of future studies will be processed locally whenever possible, though the availability of consumables remains difficult.

4. How does the sensitivity of the TS POC CC test in cohort 1 in this study compare to individuals from community-based studies (which will be a mixture of symptomatic and asymptomatic populations) in relation to lines 517 – 525?

Reply: The sensitivity of the TS POC CC test in cohort 1 (77% [38–99%]) is higher than the sensitivity observed in community-based studies in Zambia (35% [14–63%]), which predominantly involved asymptomatic populations. We revised the text for clarity:

“Sensitivity was low among participants with gastrointestinal symptoms (25% [6 – 53%]) and other symptoms (44% [2 – 95%]), similar to rates observed in asymptomatic rural communities in Zambia (35% [14–63%]) [16]. The sensitivity was higher among participants with neurological signs/symptoms (77% [38-99%], cohort 1), likely due to higher parasitic loads, resulting in elevated antibody/antigen levels…”

5. The authors should compare their findings with this POC to the POC test based on urine samples indicated by Toribio et al. 2023 and Toribio et al. 2024 (see below). Although this test is based on antigen detection, it would be important to compare the performance and potential utility of both tests: Toribio L, Handali S, Marin Y, Perez E, Castillo Y, Bustos JA, O'Neal SE, Garcia HH. A Rapid Point-of-Care Assay for Cysticercosis Antigen Detection in Urine Samples. Am J Trop Med Hyg. 2023 Feb 6;108(3):578-580. doi: 10.4269/ajtmh.22-0598. Toribio LM, Vásquez A, Castillo Y, Salas SM, Perez E, Bustos JA, O'Neal SE, Garcia HH. Concordance between a New Rapid Point-Of-Care Assay and Standard ELISA in the Detection of Cysticercosis Antigens in Urine. Am J Trop Med Hyg. 2024 Aug 13;111(4):823-825. doi: 10.4269/ajtmh.24-0171.

Reply: The paper of Toribio et al 2023 ” A Rapid Point-of-Care Assay for Cysticercosis Antigen Detection in Urine Samples” evaluated the test for NCC (using 30 patients with subarachnoid neurocysticercosis to assess sensitivity), so results are not comparable (cysticercosis vs neurocysticercosis and laboratory case control vs observational filed study). But we’ve included the reference in the discussion as follows:

“Recently, a point-of-care test for the detection of antigens in urine has undergone laboratory validation, showing promise for future field validation [32]. Urine is less invasive than blood sampling and more practical to collect than stool samples, offering potential advantages.”

6. The text in the conclusion section (lines 564 – 67): “Additionally, transforming a continuous variable, such as antibody levels or DNA concentrations, into a binary outcome (presence or absence of infection) leads to a loss of valuable information, which can result in misclassification and diminish the overall accuracy of a diagnostic test.” Would be better placed and expanded on in the discussion section.

Reply: We moved this above in the discussion section as suggested:

”The smaller-than-anticipated analysis population in this study may have affected the reliability and precision of the estimates obtained, so the results should be interpreted with caution. Moreover, transforming continuous variables, such as antibody levels or DNA concentrations, into binary outcomes (presence or absence of infection) inevitably leads to a loss of valuable information. This simplification can result in misclassification, particularly near cut-off thresholds.”
