# Supplementary material for: Evaluation of a rapid lateral flow assay for the detection of taeniosis and cysticercosis at district hospital level in Tanzania: A prospective multicentre diagnostic accuracy study
Source: PLoS Negl Trop Dis. 2025 Mar 28;19(3):e0012310. doi: 10.1371/journal.pntd.0012310 (PMC11977959; doi:10.1371/journal.pntd.0012310)
Supplement: Attachment — Submitted filename: Response to reviewers_R2.docx [file pntd.0012310.s007.docx]

**Reviewer #1:**

The authors added appendices to address previous recommendations. They also expanded the limitations in the conclusions to acknowledge the latent class definition problem.

Reply: We appreciate the reviewer's recognition of the additional appendices and the expanded discussion of limitations. Thank you for your constructive feedback to improve our manuscript.

**Reviewer #2:**

First, I would like to thank the authors for their thorough revision of the manuscript. The responses demonstrate careful consideration of the comments and substantial effort has been made to address each point raised. The expanded discussion and clarifications have notably improved the manuscript. While I understand the word limit constraints, I have concerns about the prioritisation of information in your manuscript: 1. Abstract and Methods: While you mention space constraints, I believe some critical information about the current study design should take precedence over contextual information about the larger project. Specifically:

(1) The number of diagnostic tests used per participant; (2) the analysis population sizes for BLCM. These are essential details for readers to understand and interpret your results properly.

Reply:

(1) we have included the number of tests in the abstract: “Lacking a gold standard test for both infections, diagnostic accuracy was evaluated using results of two coprological and two serological tests for taeniosis, and three serological tests for cysticercosis, in a Bayesian Latent Class Model approach.”;

(2) we have also included the number of complete cases in the abstract to provide clarity on the analysis population: “Complete case data were available for 120, 114, and 53 participants for taeniosis, and 126, 122, and 55 participants for cysticercosis.”

2. Background Section: While I appreciate that this study is part of a larger project, I maintain that the current manuscript should prioritise details relevant to this specific analysis. I suggest: (1) Moving broader project context to a single paragraph with appropriate references. These suggestions aim to ensure that readers have access to all essential information about the current study's design and analysis, while still adhering to word limits. Context about the larger project, while valuable, should not come at the expense of key methodological details.

Reply: We agree that the context is important to frame the results of this manuscript within the larger project, so the introduction section provides this context with the appropriate references:

“…To evaluate its real-world performance in resource-poor, highly endemic areas in sub-Saharan Africa, studies were designed to evaluate the performance characteristics of the TS POC test in two different settings: in rural communities in Zambia [13], and in district hospitals in Tanzania [14]. The evaluation of the TS POC test for the detection of *T. solium* taeniosis and cysticercosis at community level has been reported elsewhere [15,16]. Also the evaluation of the TS POC test for the neuroimaging-based diagnosis of NCC in both settings were reported elsewhere [17,18], and are thus out of the scope of this paper. The focus of this paper is the evaluation of the TS POC test for the detection of infection with the adult tapeworm (taeniosis) and/or the larval stage (cysticercosis) of *T. solium* in individuals attending district hospitals (Tier 2 level) in Tanzania.”

In addition, I have few remaining concerns in the methods and results section: Regarding Table 3 and Confidence Intervals: Your response suggests that calculating CIs is "not straightforward" due to the weighting in the two-stage design. However, this doesn't necessarily mean it's impossible. There are established methods for calculating confidence intervals for weighted estimates, including bootstrap methods that could account for the complex sampling design. Could you either: (1) Implement one of these methods to provide CIs, or (2) More explicitly explain why these standard approaches for weighted data would not be applicable in your specific case?

Reply:

We appreciate the reviewer’s suggestion and acknowledge the value of confidence intervals. However, incorporating them into Table 3 would significantly increase its complexity without providing substantial additional insights. Including confidence intervals for each of the three agreement measures in every cell would require either six additional columns or 18 additional rows of results, making the table cumbersome and difficult to interpret. Given that agreement between tests is an exploratory endpoint, we believe the current presentation strikes the right balance between clarity and informativeness. Additionally, we have already addressed the limitations of these estimates in the text, explicitly stating that they should be interpreted with caution due to the low number of test-positive samples. For these reasons, we believe the current format remains the most appropriate for the manuscript.

Regarding BLCM and Prior Influence: Your response about the prior influence raises several concerns: (1) Your last sentence mentions that "the analysis allowed the sensitivities and specificities to differ conditional on the other test results," but this needs more elaboration. This appears to address the question about conditional dependence, but it's not clear how this was implemented in the model (dependence in positives, dependence in negatives, dependence in both?). (2) Not all details of the BLCM and modelling diagnostics are provided. Hence, it is not possible to assess whether the right approach was adopted and to reproduce the results. I suggest that the code is included in the SI.

Reply: Thank you for your feedback. To enhance transparency and reproducibility, we included the R codes of the models in S2 Appendix, allowing specialists in BLCM to review all details. This ensures transparency while keeping the main text accessible for readers who are less familiar with BLCM.

**Reviewer #3:**

Questions/points raised have been well addressed. Thank you.

Reply: We appreciate your positive feedback and thank you for your time and constructive comments, which have helped improve the manuscript.
